# Genetic Differentiation in Insular Lowland Rainforests: Insights from Historical Demographic Patterns in Philippine Birds

**DOI:** 10.1371/journal.pone.0134284

**Published:** 2015-08-27

**Authors:** Luis Antonio Sánchez-González, Peter A. Hosner, Robert G. Moyle

**Affiliations:** Natural History Museum and Biodiversity Institute, Dyche Hall, University of Kansas, 1345 Jayhawk Blvd., Lawrence, Kansas 66045-7561, United States of America; Smithsonian Conservation Biology Institute, UNITED STATES

## Abstract

Phylogeographic studies of Philippine birds support that deep genetic structure occurs across continuous lowland forests within islands, despite the lack of obvious contemporary isolation mechanisms. To examine the pattern and tempo of diversification within Philippine island forests, and test if common mechanisms are responsible for observed differentiation, we focused on three co-distributed lowland bird taxa endemic to Greater Luzon and Greater Negros-Panay: Blue-headed Fantail (*Rhipidura cyaniceps*), White-browed Shama (*Copsychus luzoniensis*), and Lemon-throated Leaf-Warbler (*Phylloscopus cebuensis*). Each species has two described subspecies within Greater Luzon, and a single described subspecies on Greater Negros/Panay. Each of the three focal species showed a common geographic pattern of two monophyletic groups in Greater Luzon sister to a third monophyletic group found in Greater Negros-Panay, suggesting that common or similar biogeographic processes may have produced similar distributions. However, studied species displayed variable levels of mitochondrial DNA differentiation between clades, and genetic differentiation within Luzon was not necessarily concordant with described subspecies boundaries. Population genetic parameters for the three species suggested both rapid population growth from small numbers and geographic expansion across Luzon Island. Estimates of the timing of population expansion further supported that these events occurred asynchronously throughout the Pleistocene in the focal species, demanding particular explanations for differentiation, and support that co-distribution may be secondarily congruent.

## Introduction

Widespread lowland rainforest bird species are of great interest for studying phylogeographic structure, which links genetics and geography [1, 2, 3, 4]. In regions where lowland rainforest has fewer putative geographic/ecological barriers, species may be expected to be widespread, with limited phylogeographic structure [5, 6, 7]. However, many bird species exhibit strong genetic differentiation across seemingly continuous lowland forests, suggesting that current or past landscape features restrict gene flow between populations [8, 9, 10, 11, 12, 13, 14, 15]. In the naturally fragmented insular landscapes of Southeast Asia, widespread lowland rainforest species often show extensive phylogeographic structure associated with repeated cycles of connection and isolation of island complexes across the region during Quaternary climatic changes [16, 17, 18, 19, 20]. However, the possibility of isolation and genetic differentiation within continuous lowland forests has received relatively little attention compared to isolation across marine barriers (but see [21, 22] and references therein).

Island systems offer advantages for evolutionary studies because of their unique properties (e.g. [23, 24]), which have promoted both adaptive radiations (e.g. [25, 26, 27, 28, 29]), and high levels of endemism (e.g., [30, 31, 32,]). However, in island birds, allopatric differentiation between populations on separate islands is thought to be the dominant mode of speciation (e.g., [33, 34]). Evidence from other seemingly less-vagile vertebrates, however, suggests that speciation may occur within a single island. Recent studies have shown this phenomenon to be particularly pervasive in groups such as amphibians (e. g. [35, 36]), reptiles (e. g. [37, 38, 39, 40]), and small mammals (e.g. [41, 42, 43]). For birds, intra-island speciation has been suggested to occur mainly in large, topographically complex islands (>100,000 km^2^, e.g. [21, 34, 44], but see [45]). However, intra-island speciation in apparently continuous habitats, such as lowland rainforests, may be overlooked because taxa showing diagnostic morphological differences are generally treated either as subspecies or as part of geographic clines on the basis of habitat continuity and potential reproductive links (e.g. [29]).

The Philippine archipelago provides a natural model to test for genetic differentiation in continuous habitats within islands. With the exception of Palawan and some of its offshore islands which were apparently united to the Sunda Shelf (reviewed in [46]), the Philippine archipelago is oceanic in origin (reviewed in [47]). After its origin by complex geological activity, the archipelago was subjected to climatic and sea-level changes in the Pleistocene [48, 49] that produced cycles of isolation and aggregation of islands into larger landmasses, providing opportunities for both dispersal and isolation [48]. These Pleistocene Aggregate Island Complexes, or PAICs [49] were apparently never joined to other PAICs, because they were separated by deep water channels (>120 m, [48]).

**Fig 1:**
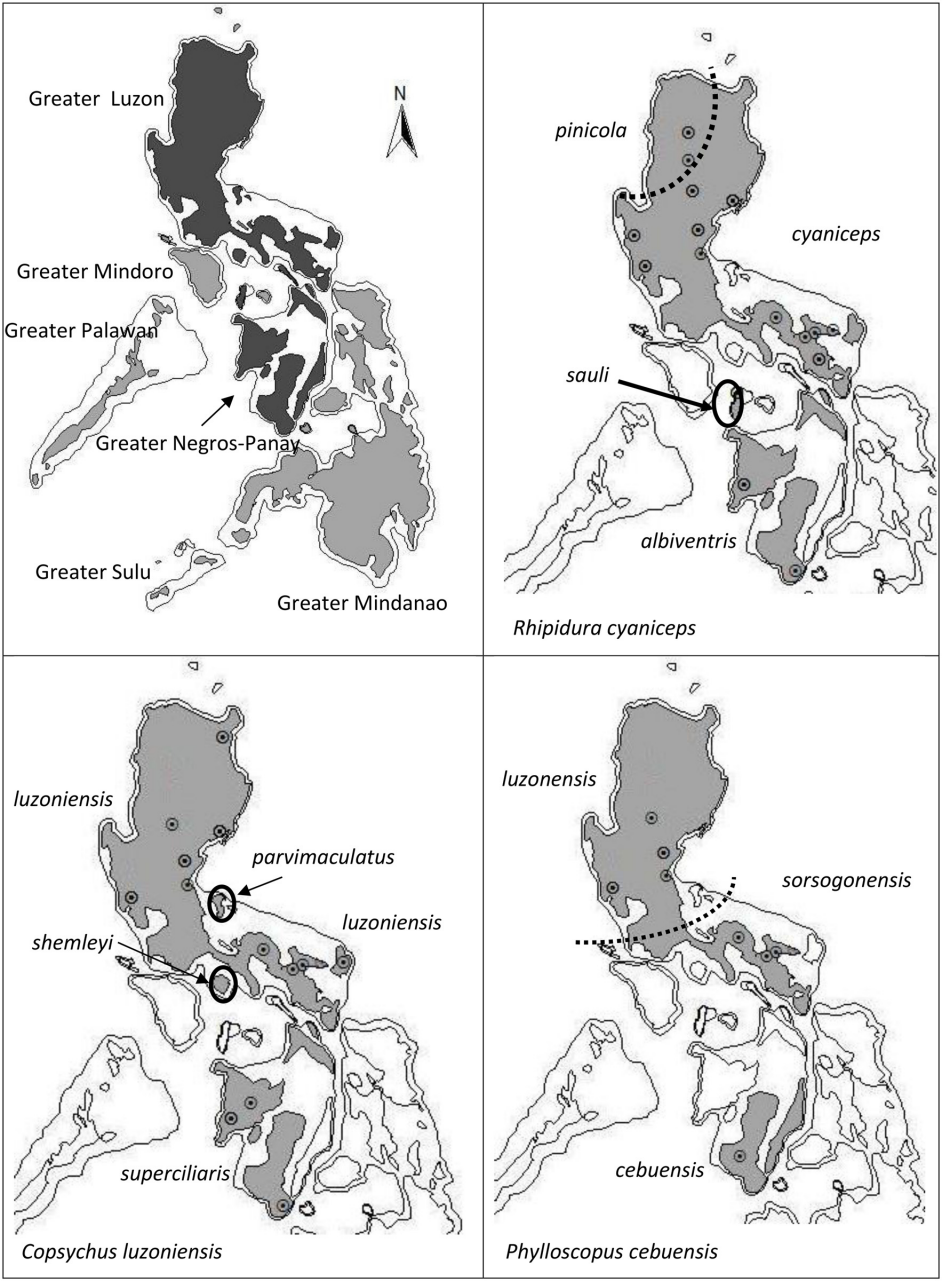
Map of the Philippines showing the limits to Pleistocene Aggregates Island Complexes PAIC (outline) and emerged land (shaded) based on Heaney (1985), study areas are in dark grey. Dotted circles represent sampling localities (See S1 Table for details). Maps show distribution for each taxa, and the subspecies described (Dickinson et al. 1991). Dashed lines represent putative borders for currently accepted subspecies in Luzon.

The dominant paradigm for explaining the high diversity levels in the Philippines has been based on the arrangement of exposed land among aggregated islands during Pleistocene sea-level changes (reviewed in [32]), predicting that most speciation events among PAICs are due to vicariance [16]. Recent work, however, has shown that the PAIC paradigm may not explain many observed diversity patterns (reviewed in [32]), and that dispersal or within-PAIC differentiation may have played a more important role than expected. We tested the prevalence of the PAIC paradigm in three primarily lowland rainforest passerine birds: Blue-headed Fantail (*Rhipidura cyaniceps*, Rhipiduridae), White-browed Shama (*Copsychus luzoniensis*, Turdidae) and Lemon-throated Leaf-Warbler (*Phylloscopus cebuensis*, Phylloscopidae). These taxa are co-distributed within the Greater Luzon PAIC, which includes the present-day islands of Luzon, Polillo, Catanduanes, and Marinduque; and in the Greater Negros-Panay PAIC, which includes Negros, Panay, Cebu, Ticao, and Masbate islands. Each species is common in forested habitats on these islands, and they are regularly found together in mixed species flocks. Both inter- and intra-island plumage differences have been documented in these species, as suggested by recognized subspecies throughout their range (Fig 1, [29, 31]). In each species, multiple described subspecies are endemic to Greater Luzon (two in *R*. *cyaniceps* and *P*. *cebuensis*, three in *C*. *luzoniensis*), and single subspecies are endemic to Greater Negros-Panay.

**Fig 2:**
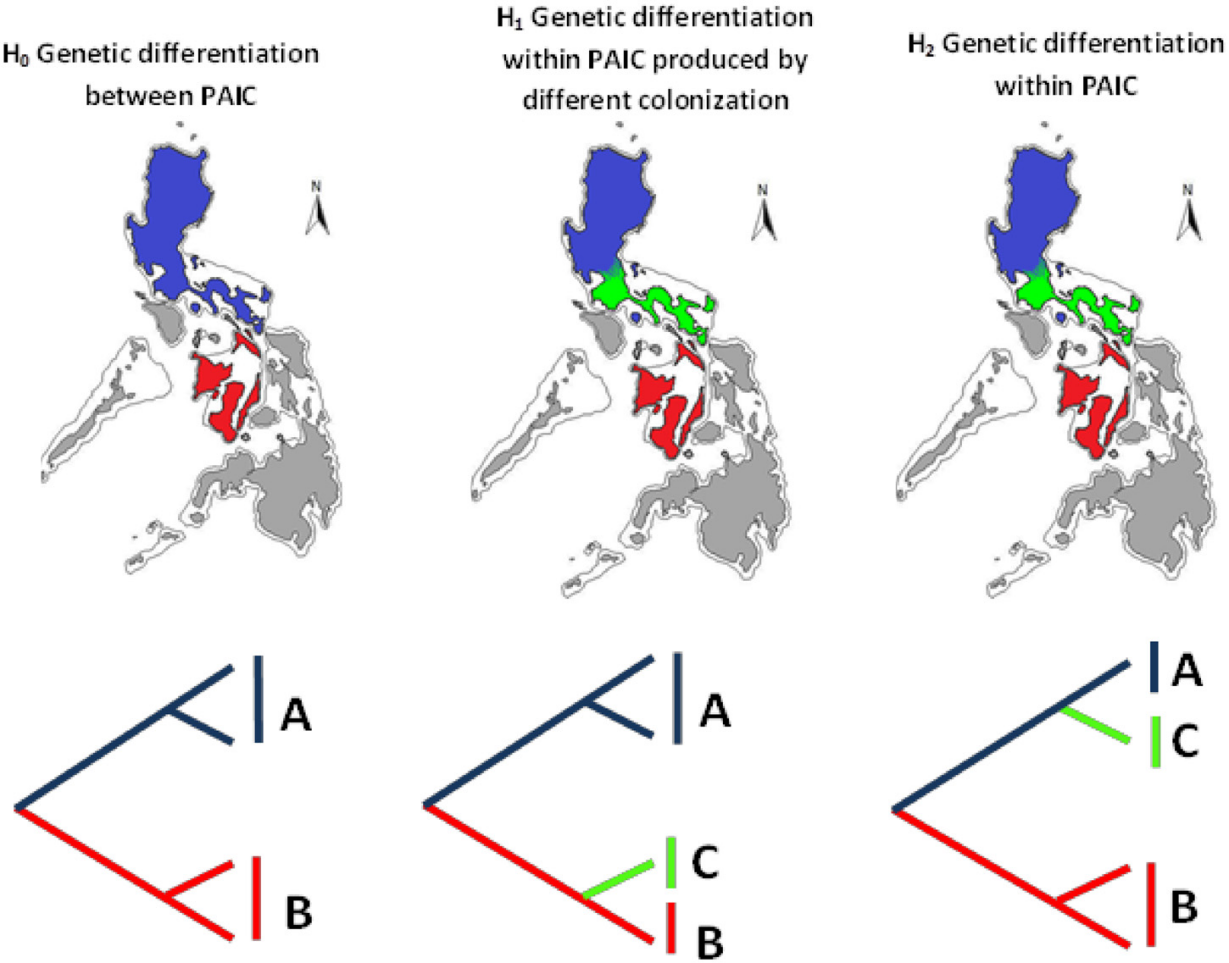
The set of hierarchical hypothesis tested in this study, with their geographic representation and phylogenetic expec. Blue: Greater Luzon, Red: Greater Negros-Panay; green: Bicol Peninsula. Areas in light grey were not considered.

We used tools from phylogenetics and phylogeography to estimate historic demographical patterns in these co-distributed Philippine passerines. Given the extensive effects of Pleistocene climatic changes in Southeast Asia [48, 50, 51, 52, 53], we may expect substantial changes in their geographic distribution, and consequently, that some populations may have experienced demographic changes. However, the effects of Pleistocene climatic changes in the oceanic Philippines remain little studied; thus, phylogeographic approaches may be of great utility in proposing primary hypotheses about the evolutionary history of the Philippine biota. Results were used for discriminating between different scenarios likely responsible for producing phylogeographic structure, such as the PAIC paradigm [48]. Based on a previous study that documented within-PAIC differentiation [54], we tested the following hierarchical biogeographic hypotheses for the co-distributed species in this study (Fig 2): a null hypothesis (H_0_) corresponding to the classical PAIC paradigm, in which genetic differentiation is expected to be partitioned between clades restricted to PAICs; alternative hypotheses involving genetic differentiation as a result of different colonization events, resulting in unrelated clades in a single PAIC (H_1_), and within-island genetic differentiation, in which sister clades are distributed in a single PAIC (H_2_).

## Materials and Methods

### Taxon sampling

The three focal species are endemic to the Greater Luzon + Greater Negros-Panay PAICs. In addition to these islands *R*. *cyaniceps* is also found on Tablas Island (Romblon Island Group). Neither *R*. *cyaniceps* nor *C*. *luzoniensis* are present on Cebu (Fig 1). Of the total samples, 40 corresponded to *Copsychus luzoniensis*, 28 to *Phylloscopus cebuensis* and 50 to *Rhipidura cyaniceps* (S1 Table, see Acknowledgements). To ensure thorough assessment of differentiation patterns within Greater Luzon, we sequenced multiple individuals from as many localities as possible. A Gratuitous Permit (GP) to conduct research and collect specimens in all sampling localities was issued by Mundita S. Lim, Director of the Biodiversity Management Bureau (BMB), Republic of the Philippines. Field studies did not involve endangered or protected species. Birds were captured using mist-nets. Nets were checked every hour, with birds immediately released if not needed for future study. Birds were euthanized via thoracic compression or isoflurane open-drop. This project operated under the University of Kansas Animal Care and Use Committee (IACUC approval AUS no. 174–01), issued to R.G.M. at the University of Kansas.

Because species-level relationships of *R*. *cyaniceps* and *C*. *luzoniensis* have been studied previously, outgroup choice was straightforward. Species selected as outgroups for *C*. *luzoniensis* were *Copsychus niger* from Palawan Island, Philippines, and *Copsychus malabaricus*, a widely distributed species in southeastern Asia [19, 20]. Outgroup selection for *R*.*cyaniceps* relied on two recent studies [55,56], showing that the three traditionally recognized endemic Philippine *Rhipidura* (including *cyaniceps*) are a monophyletic group, thus we included samples of the other two endemic Philippine species. Finally, because the monophyly of *P*. *cebuensis* has been questioned, with some authors lumping this species and *P*. *olivaceus* into a single taxon [57, 58], we included samples of the latter, which is likely the sister species of *P*. *cebuensis* [59]. We also included samples of *P*. *ijimae* and *P*. *coronatus* as additional outgroups [60].

### DNA sequencing

DNA was extracted from frozen tissue using Proteinase K digestion procedures following the manufacturer’s protocols (DNeasy; Qiagen, http://www.qiagen.com/). Markers selected for this work have been used widely in bird systematics and biogeography, and include the entire second subunit (ND2, 1041 bp) and third subunit (ND3, 351 bp) of nicotinamide adenine dinucleotide dehydrogenase. These markers were amplified with the external primers L5215 and H6313 for ND2 [61] and L10755 and H11151 for ND3 [62], as well as the ND2 internal primers 487L (provided by C. Oliveros C., unpublished), ND2-SWH [63], and H5766 [61].

To obtain independent assessments of phylogenetic relationships, we also sequenced three nuclear markers: myoglobin intron 2 (Myo2, 541bp), glyceraldehyde-3-phosphodehydrogenase intron 2 (G3pdh, 358 bp), and beta-Fibrinogen intron 5 (FIB5 565 bp). The *Copychus* and *Phylloscopus* nuclear datasets included Myo2 and G3pdh, but not FIB5, whereas the *Rhipidura* nuclear dataset included FIB5 only. Amplification and sequencing of FIB5 used the primers FIB5 and FIB6 [64] as well as the internal primers FIB5F2 and FIB6R2 [65]; G3pdh used primers G3p13a and G3p13b [65, 66] and Myo2 used the primers Myo2 and Myo3F [67, 68], as well as the internal primers 340R, MyoIntR and MyoINTF [68].

Genomic DNA was amplified using 5-primeTaq DNA polymerase under standard PCR thermocycling protocols and visualized in agarose gels stained with ethidium bromide. Resulting products were cleaned with ExoSAPIT (GE Healthcare Corp.) and the purified products were cycle-sequenced with ABI Prism BigDye v3.1 terminator chemistry. Cycle-sequenced products were purified with ethanol precipitation, and sequenced on an ABI 3730 automated sequencer. Sequences were aligned using MAFFT [69], as implemented in Geneious 7.0.2 [70]. Alignments for each gene were further inspected by eye, and insertions and deletions (indels) were adjusted as necessary. Heterozygous positions in the nuclear introns, determined by double-peaks of similar height, were coded following the IUPAC ambiguity codes.

### Phylogenetic Analyses

Phylogenetic relationships were reconstructed from the concatenated dataset for each taxon through Maximum Likelihood analysis (ML) as implemented RAxML 7.0.3 [71], which allows for different sequence evolution models to be incorporated into the analysis; nodal support was assessed via non-parametric bootstrapping [72] with 1000 replicates. We also used Bayesian Inference (BI) on the complete dataset for each species using MrBayes 3.2. [73]. Each dataset was partitioned by gene and codon positions for the nuclear intron and mitochondrial genes respectively [74, 75]. The Akaike Information Criterion (AIC), as implemented in MrModeltest [76], was used to determine the best substitution model for each partition. BI was implemented for 10^7^ generations and sampled every 500 generations. Stationarity of the MCMC chains was assessed in Tracer v1.5.0 [77], after which the first 30% generations were discarded as initial burn-in. All remaining trees in the summary were used to produce a single 50% majority-rule consensus tree. In order to ensure that examination of tree space was appropriate, topological convergence was assessed in the on-line application AWTY [78] by using the compare function, which plots posterior probabilities of all splits for paired MCMC runs. Inspection for stationarity revealed that parameter and topological space were searched thoroughly.

### Population genetic parameters

All population parameters were estimated from the mitochondrial dataset (ND3 and ND2) for each species. Genetic diversity was assessed using indices of haplotype diversity (Hd) and nucelotide diversity (π), with samples grouped by present-day island boundaries (Fig 1).

Following the phylogenetic results, Luzon samples were further divided according to the obtained clades, corresponding to northern Luzon and the Bicol Peninsula. However, the geographic structure in these clades showed the Zambales region (in western Luzon) samples grouped with the northern Luzon clade in both *R*. *cyaniceps* and *P*. *cebuensis*, but with the Bicol Peninsula clade in *C*. *luzoniensis*. These arrangements suggest particular dynamic biotic processes in the Zambales region, for which we conducted separated analyses for the populations of the three species. As a relative measure of divergence between populations, we estimated Nei’s genetic distance values (D*xy*), using DNAsp v5 [79] with a Jukes-Cantor correction [80], between groups of populations identified in the phylogenetic results.

Genetic structure in the three species was explored using three-way AMOVA and *Fst*. For the AMOVA analysis, samples were arranged in groups corresponding to the clades found in the phylogenetic analyses. The significance of the AMOVA results was assessed through 10,000 non-parametric permutations. The parameter *Fst* was calculated through pairwise differences between haplotypes; significance of the *Fst* parameter was also assessed with 10,000 permutations. Finally, as an additional measure of gene flow, we calculated the number of migrants per generation (Nm). Because some studies have shown that populations of *R*. *cyaniceps* and *C*. *luzoniensis* in the Greater Negros-Panay PAIC and the Romblon Island Group are evolutionarily distinct from those in Greater Luzon [20, 55, 81], AMOVA analyses were repeated using the same parameters as described above but excluding populations in these island groups. All statistics were calculated in Arlequin ver. 3.5.1.3 [82]. Interpretation of *Fst* and Nm values followed the guidelines in Hartl and Clark [83].

We tested whether populations of the three species had experienced demographic changes by calculating Fu’s *F*_*s*_ statistic [84], which indicates whether individual populations are evolving according to the Wright-Fisher model. Significance of Fu’s F_s_ was determined using a p-value of 0.02 as suggested by Fu [84]. We also calculated Tajima’s *D* statistic [85], another measure of the selective neutrality of markers in a population. Significance of Fu’s F_s_ and Tajima’s *D* were calculated by constructing 1000 coalescent simulations in DnaSP v5 [79].

Population history was further inferred by plotting mismatch distributions [86, 87] and calculating their significance using Ramos-Onsins and Rozas R_2_, which is better suited for small sample sizes [88]. R_2_ significance was estimated through 1000 coalescent simulations for the different clades (and the Zambales subpopulation) of each species in DnaSP v5 [79]. For clades showing significant population growth, the parameter Tau (τ) was used to calculate the time *t* of potential step-wise expansion from a relatively small, but constant population to a large population of size Ɵ_1_ over *t* generations in the past, with *t* = τ/2u, where τ = age of expansion (in mutational units) and u = 2μk, where μ = mutation rate and k = the length of the sequence [87]. We used mutation rates of 1 x 10^−9^ substitutions/site/year (s/s/y), which is supported by a large dataset of studies on passerine birds [89], 2.7 x 10^−9^ s/s/y, which is a ND2 specific mutation rate calculated from mockingbirds in the Galapagos Islands [90], and a faster rate of 4 X10^-9^ s/s/y, which accounts for uncertainties about mitochondrial substitution rates [91, 92]. Some researchers have suggested that the estimation of τ according to Rogers [93] often leads to an underestimation of the age of expansion, due to the omission of heterogeneity in mutation rates among sites [94]. Thus, we calculated τ values by applying a least-squares approach, as implemented in Arlequin ver. 3.5.1.3 [82]. Confidence intervals for τ were calculated using 3000 parametric bootstrap replicates [94], as implemented in Arlequin ver. 3.5.1.3 [82].

Finally, because it has been suggested that bifurcating trees may not always fully represent intraspecific phylogenies due to the coexistence of ancestral and derived haplotypes in a given sample [95], we also estimated Median-joining networks using a median-joining method [96] in Networks 4.6.0.0 (http://www.fluxus-engineering.com), assigning equal weights to all variable sites and with default values for the epsilon parameter (Ɛ = 0).

## Results

### Phylogenetics

In all, 1967 characters were included in the complete dataset for *R*. *cyaniceps*, 2198 characters for *C*. *luzoniensis*, and 2429 characters for *P*. *cebuensis*. Sequences for *R*. *cyaniceps*, *C*. *luzoniensis*, and *P*. *cebuensis* are deposited in GenBank (S1 Table). Sequence characteristics for each gene partition and the selected models of evolution are provided in S2 Table.

**Fig 3:**
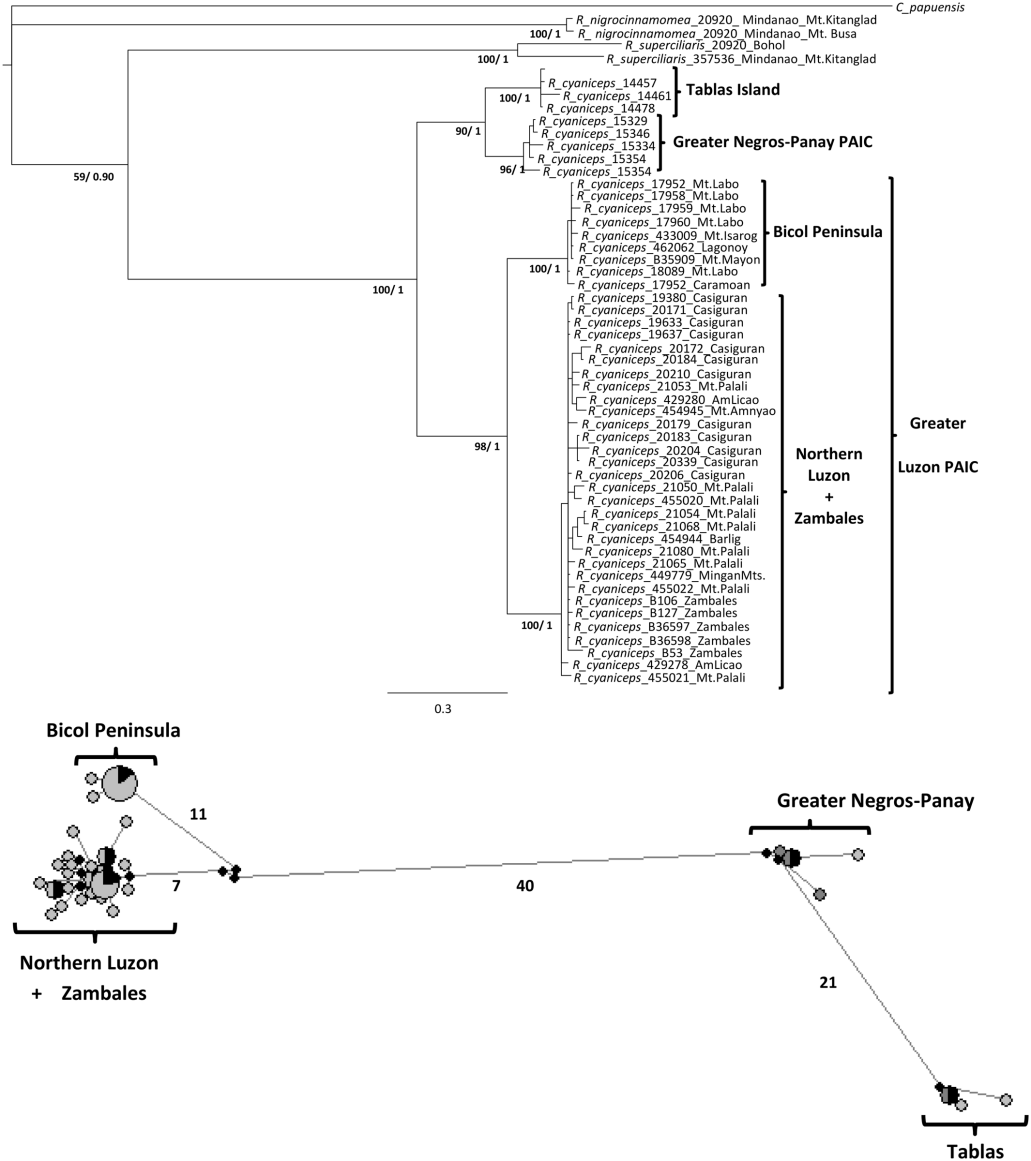
Maximum Likelihood tree and median-joining haplotype network of the mtDNA dataset for *R*. *cyaniceps*. Numbers in the branches refer to bootstrap support for the ML (before slash) and posterior probabilities from the BI (after slash). In the Median-joining haplotype network each ellipse represents a unique haplotype; different sizes and shading (a black shaded portion represents an individual sharing that haplotype) according to the frequency of occurrence. Each line connecting haplotypes represent a single mutational step. Numbers along lines indicate two or more steps separating haplotypes. Small open circles represent missing (unsampled) haplotypes.

**Fig 4:**
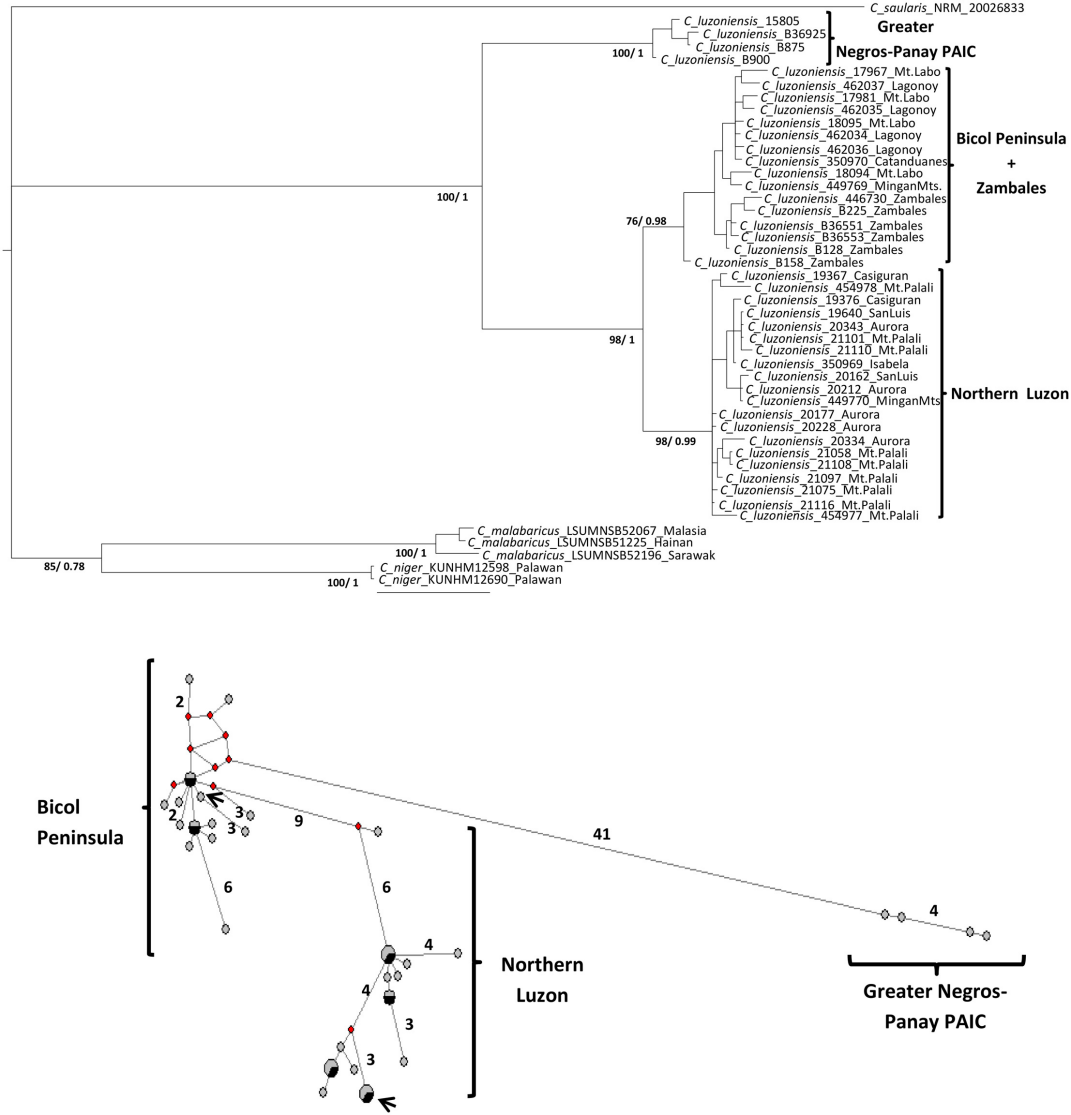
mtDNA phylogram of *C*. *luzoniensis*. Numbers in the branches refer to bootstrap support for the ML (before slash) and posterior probabilities from the BI (after slash). Median-joining haplotype network for the mtDNA dataset. Arrows signal haplotypes found in the Mingan Mountains, eastern Luzon. See Fig 3 for details.

**Fig 5:**
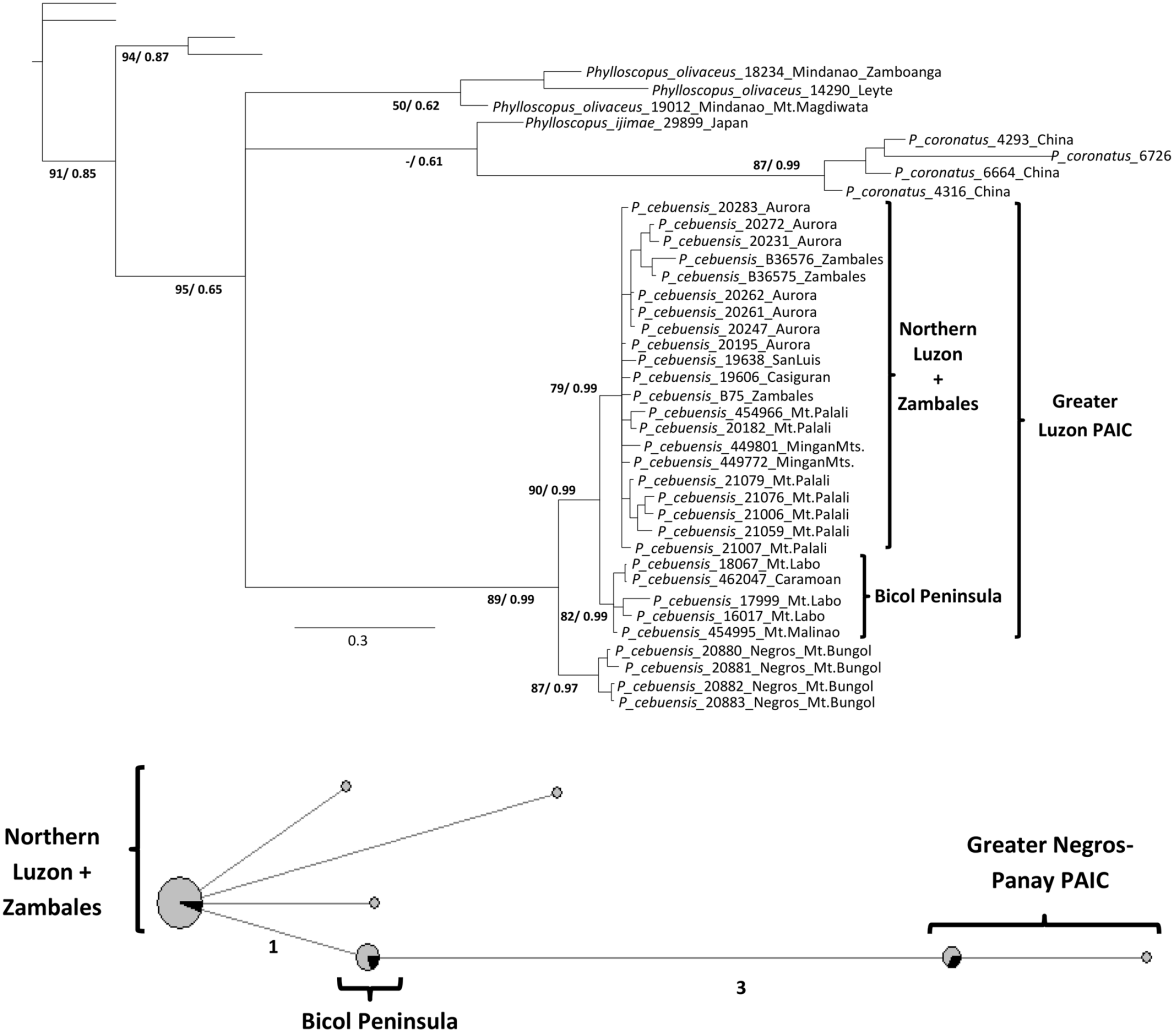
mtDNA phylogram of *P*. *cebuensis*. Numbers in the branches refer to bootstrap support for the ML (before slash) and posterior probabilities from the BI (after slash). Median-joining haplotype network for the mtDNA dataset. See Fig 3 for details.

Phylograms from the mitochondrial DNA (mtDNA) dataset were identical to those obtained from the complete dataset in *R*. *cyaniceps* (Fig 3, see [55]), as well as for *C*. *luzoniensis* (Fig 4) and *P*. *cebuensis* (Fig 5). Nuclear phylograms (nDNA) for each species showed two sister clades including all of the Greater Luzon PAIC samples and Greater Negros-Panay PAIC (Negros and Panay islands) samples. However, no structure in the nDNA datasets was apparent within Greater Luzon, likely due to the fourfold higher effective population size, longer coalescence times, male biased dispersal, and slower mutation and rates of nDNA markers, consistent with expectations from coalescent theory [97].

ML and BI results were largely congruent in all three cases (Figs 3, 4, and 5). All analyses showed each of the three taxa as monophyletic. In each case, there were three relatively well-supported clades: a Western Visayas clade (Greater Negros-Panay PAIC), and two clades from Greater Luzon, corresponding to northern and southern parts of the island. Samples from the Zambales region in western Luzon were embedded within the northern Luzon clade in *R*. *cyaniceps* and *P*. *cebuensis*, but with the Bicol Peninsula clade (including Catanduanes Island) in *C*. *luzoniensis*. One additional monophyletic grouping included the Tablas island samples of *R*. *cyaniceps*, as previously reported [55].

### Phylogeography

Genetic structure (Table 1) analyses revealed high values of differentiation between PAICs. High values for Nei’s corrected distance (Dxy) were found for comparisons between Greater Luzon PAIC and Greater Negros-Panay PAIC for *R*. *cyaniceps* and *C*. *luzoniensis*, consistent with recent systematic treatments in which these populations have been recognized as full species (*R*. *albiventris* and *C*. *superciliaris*, respectively, [20, 55, 81, 98]). Divergence also was found within PAIC boundaries, as in Greater Luzon PAIC, where values of genetic flow and genetic differentiation are also high [82]. High levels of haplotype diversity (Hd) and low levels of nucleotide diversity (π) were observed in the populations of the three species (Table 2), suggesting rapid population growth according to Category 2 in Grant and Bowen [99].

**Table 1:** Gene flow parameters estimated for the three lowland Passerines under study. Above the diagonal in each species, are *Fst* (Nm); below the diagonal, values of Dxy (Da), where Dx indicates the average number of nucleotide substitutions per site between populations (percentage), and Da indicates the average number of net nucleotide substitutions per site between populations (Nei 1987).

		N Luzon	Zambales	Bicol Pen	Visayas
**Taxon**	***Rhipidura cyaniceps***				
Clade	**N Luzon**	**-**	0.05921* (4.11)	0.85980 (0.04)	0.92014 (0.05)
	**Zambales** ^**1**^	0.092 (0.037)	-	0.95550 (0.02)	0.93976 (0.03)
	**Bicol Peninsula**	3.135 (2.881)	2.993 (2.886)	-	0.96732 (0.03)
	**Visayas**	4.960 (4.535)	4.980 (4.665)	4.638 (4.373)	-
**Taxon**	***Copsychus luzoniensis***				
Clade	**N Luzon**	**-**	0.71289 (0.09)	0.73042 (0.17)	0.89520 (0.04)
	**Zambales** ^**1**^	3.333 (2.839)	-	0.20805 (0.89)	0.92532 (0.04)
	**Bicol Peninsula**	2.437 (1.831)	0.827 (0.298)	-	0.91109 (0.04)
	**Visayas**	6.607 (6.084)	6.027 (5.844)	5.772 (5.318)	-
**Taxon**	***Phylloscopus cebuensis***				
Clade	**N Luzon**	**-**	-0.19531* (0.0)	0.72870 (0.18)	0.89845 (0.06)
	**Zambales** ^**1**^	0.054 (0.00)	**-**	1.00000 (0.0)	0.92982 (0.03)
	**Bicol Peninsula**	0.403 (0.295)	0.289 (0.289)	-	0.93363 (0.09)
	**Visayas**	1.505 (1.356)	1.235 (1.163)	1.245 (1.064)	-

* Not significant at P < 0.05

^1^ Not actually a clade (See Methods)

**Table 2:** Molecular diversity and tests of neutral evolution for the three species in this study, grouped by region and clade.

	No.	h	Hd ± SD	π ± SD	R_2_	Tajima’s *D*	Fu’s F _s_
*Rhipidura cyaniceps*							
All	49	33	0.969 ± 0.014	0.02326 ± 0.00322			
N Luzon	26	22	0.988 ± 0.014	0.00419 ± 0.00041	0.0596**	**-1.7389** *	**-15.5073** ***
Zambales	5	3	0.7 ± 0.218	0.00207 ± 0.00103	0.40000	-1.1455^1^	3.0225
Bicol Peninsula	9	5	0.722 ± 0.159	0.00230 ± 0.00120	**0.1361** *	**-1.7278** *	-1.7836
Visayas	5	5	1 ± 0.126	0.00457 ± 0.00123	0.19153	-0.9978	-1.1125
*Copsychus luzoniensis*							
All	40	31	0.985 ± 0.010	0.02139 ± 0.00314			
N Luzon	20	13	0.947 ± 0.03	0.00612 ± 0.00074	0.0912	-1.1237	-1.6843
Bicol Peninsula	10	9	0.978 ± 0.054	0.00585 ± 0.00108	**0.0784** ***	-0.09830	0.20439
Zambales	6	6	1 ± 0.096	0.00445 ± 0.00194	0.2493	-1.3152	-1.8546
Visayas	4	4	1 ± 0.177	0.00578 ± 0.00154	0.2016	0.2616	0.0432
*Phylloscopus cebuensis*							
All	30	7	0.618 ± 0.091	0.0043 ± 0.00105			
N Luzon	18	10	0.81 ± 0.093	0.00125 ± 0.00029	**0.0733** ***	**-1.9079** *	**-6.0145** ***
Zambales	3	1	-	-		**-**	
Bicol Peninsula	5	3	0.7 ± 0.218	0.00189 ± 0.00075	0.2630	-0.6682	1.0900
Visayas	4	3	0.833 ± 0.222	0.00169 ± 0.00053	0.2732	0.6501	0.3596

No., number of samples; h, number of haplotypes; Hd, Haplotype diversity and standard deviation; π, Nucleotide diversity and standard deviation; R_2,_ Ramos-Onsins and Rozas.

* P>0.05

** P>0.001

*** P>0.00001

^1^ P = 0.058

**Fig 6:**
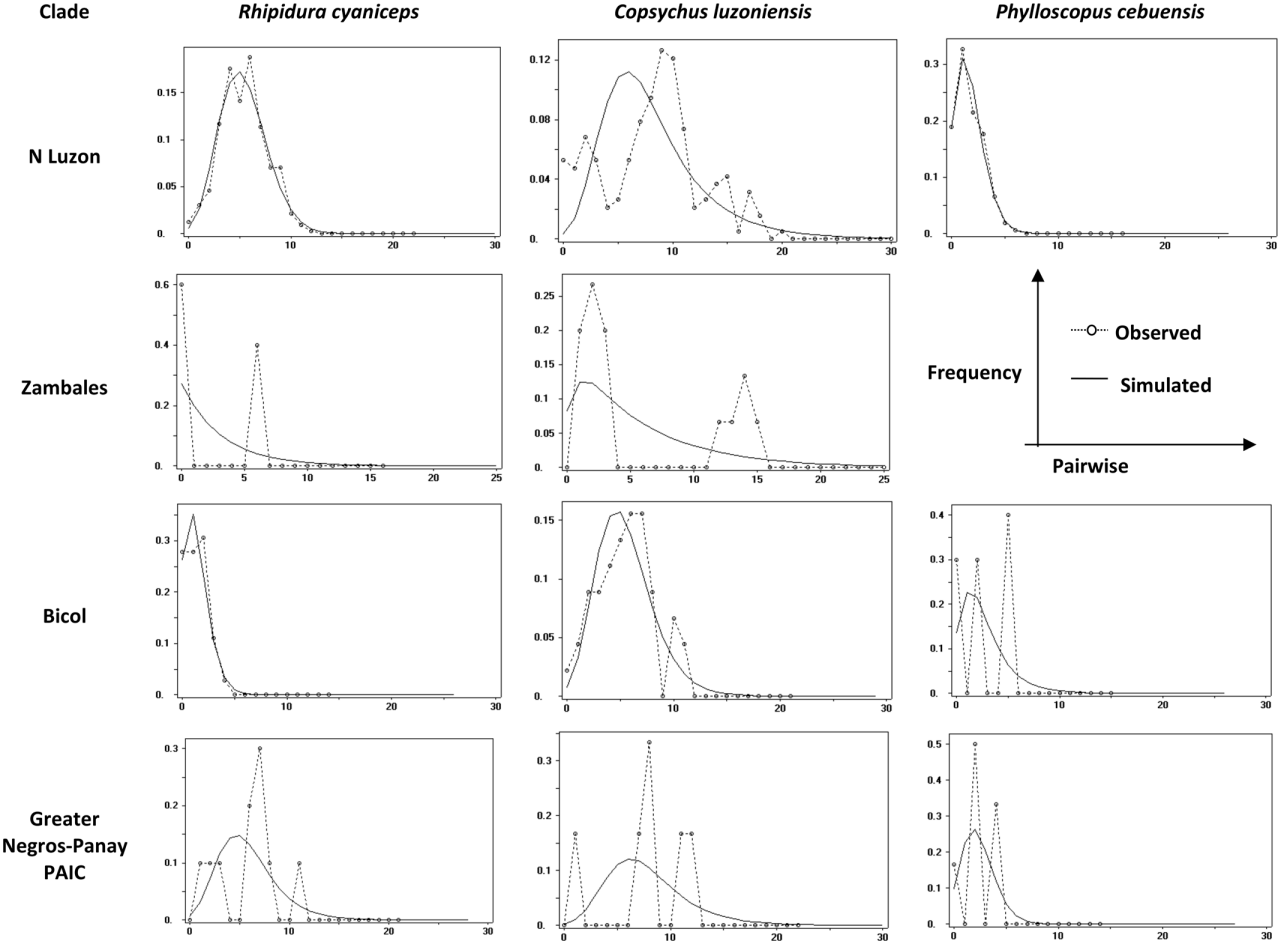
Mismatch distributions for each focal taxon. Dots represent the observed mismatch distributions, the continuous line represent the expected mismatch distributions. Due to sample size, no mismatch graph was calculated for *P*. *cebuensis* from the Zambales region in Greater Luzon PAIC. * N Luzon + Zambales for *R*. *cyaniceps* and *P*. *cebuensis*; Bicol Peninsula + Zambales in *C*. *luzoniensis*.

Mismatch distributions (Fig 6) and Ramos-Onsins and Rosas´R_2_ values (Table 2) showed similar patterns with respect to geographic areas across species. In northern Luzon, mismatch distributions for *R*. *cyaniceps* and *P*. *cebuensis* showed a unimodal pattern, suggesting population expansion; R_2_ values were significant in both species. In *C*. *luzoniensis*, a ragged pattern is apparent, suggesting that populations may be at demographic equilibrium. This same unimodal pattern is also apparent in Bicol Peninsula populations of *R*. *cyaniceps* and *C*. *luzoniensis*, in which significant R_2_ values were obtained for both species, suggesting recent population expansion in the area. For *P*. *cebuensis*, the Bicol Peninsula clade showed a multimodal pattern, suggesting persistence through small and isolated populations or a population bottleneck [100]. The Zambales populations of both *R*. *cyaniceps* and *C*. *luzoniensis* (small sample size in *P*. *cebuensis* prevented analysis) showed a bimodal pattern, suggesting either that for both species, there has been a relatively constant effective population size in the past [100], or probably, an admixture of distinct populations, which may be expected as both of these subpopulations are part of larger genetic groups located either in Northern Luzon and the Bicol Peninsula, respectively. Finally, the Greater Negros-Panay in *R*. *cyaniceps* and *C*. *luzoniensis* showed a multimodal pattern, suggesting either population bottleneck or persistence of small and isolated populations, which seems highly probable as this PAIC is presently partitioned in smaller islands fragments, in comparison to Greater Luzon PAIC. However, these results are only preliminary and should be taken with caution, given the sample size and geographic coverage, which prevented conclusive demographic inferences, especially for *P*. *cebuensis*.

Three-way AMOVA results (Table 3) suggested that the greatest variation in genetic structure of the three species is found among groups, which correspond to clades obtained in the phylogenetic analysis and PAIC distribution limits. High *Fst* values [83] were obtained in all comparisons involving populations from Greater Negros-Panay PAIC and populations in Greater Luzon PAIC (Table 2), as predicted by the PAIC paradigm [49]. However, high and significant *Fst* values (>0.72) were also obtained for within-PAIC comparisons, particularly involving Northern Luzon and the Bicol Peninsula populations in the three species. Values of *Fst* from populations in Zambales and Northern Luzon showed no significant genetic structure in *R*. *cyaniceps* and *P*. *cebuensis*, but were moderate (0.21) for *C*. *luzoniensis* populations from the Zambales and Bicol Peninsula. Gene flow was low [83] among most populations within the three focal species (Table 1), suggesting less than one migrant per generation. However, gene flow was more prevalent between populations of *R*. *cyaniceps* in northern Luzon and the Zambales (4.11 migrants per generation), and between populations of *C*. *luzoniensis* from Zambales and Bicol (1 migrant per generation). Population expansion in Northern Luzon was supported by significantly negative values obtained for Fu’s *F*_*s*_ [84] in both *R*. *cyaniceps* and *P*. *cebuensis* (Table 2). Remaining populations may have experienced population growth; however, small sample sizes may influence statistical power to detect them. Tajima’s *D* values [84] corroborated the results obtained with the Fu’s F statistic, also suggesting population expansion or stabilizing selection [99]. Additionally, the Bicol Peninsula clade of *R*. *cyaniceps* showed significant negative values, suggesting also demographic expansion in this population.

**Table 3:** AMOVA values estimated for the lowland passerines under study.

Source of variation	Sum of squares	Variance components	% of variation
***Rhipidura cyaniceps***			
Among groups	515.241	18.82169	91.06*
Among populations within groups	3.066	0.16495	0.80
Within populations	74.020	1.68227	8.14
Total	592.327	20.66892	
***Copsychus luzoniensis***			
Among groups	224.704	5.11438	35.29*
Among populations within groups	65.813	6.85668	47.32
Within populations	90.733	2.52037	17.39
Total	381.250	14.49143	
***Phylloscopus cebuensis***			
Among groups	16.974	1.21784	89.27*
Among populations within groups	0.032	-0.02769	-2.03
Within populations	4.528	0.17415	12.76
Total	21.533	1.36430	

*Not significant at P < 0.05

Although estimated population expansion dates are heavily dependent on the mutation rates used, all of them and their respective confidence intervals fall within the Pleistocene (Table 4). Median population expansion times using the standard 2% [89] rate support early Pleistocene for *R*. *cyaniceps* (1.5 mya) and early to middle Pleistocene for *P*. *cebuensis* (0.8 mya). In the Bicol Peninsula, estimates of population expansion strongly suggest two different events. For *C*. *luzoniensis*, estimates suggest mid to early Pleistocene events, whereas populations of *R*. *cyaniceps* may have expanded in the late Pleistocene.

**Table 4:** Estimated population expansion dates for the three taxa in Luzon Island. Values are given for subpopulations in either NC Luzon (*R*. *cyaniceps* and *P*. *cebuensis*) or Bicol Peninsula (*C*. *luzoniensis*) that showed evidence of population expansion (see text). Columns indicate values of τ and three age estimates along with their 95% confidence intervals. Rates based on: ^1^Weir and Schluter 2008, ^2^Arbogast et al. 2006, and ^3^Lim et al. 2011. Mutation rates in Myr^-1^.

Taxon Population	τ		μ = 0.01_1_	μ = 0.027_2_	μ = 0.04_3_
*Phylloscopus cebuensis*					
N Luzon	Estimated	3	1,077,586	399,106	269,396
	Lower bound	0.375	134,698	49,888	33,674
	Upper bound	4.078	1,464,798	542,518	366,199
	Median	2.322	834,051	308,908	208,512
*Copsychus luzoniensis*					
Bicol Peninsula	Estimated	4.73828	1,701,968	551,165	372,036
	Lower bound	1.844	662,356	245,317	165,589
	Upper bound	7.057	2,534,841	938,830	633,710
	Median	4.5	1,616,379	598,659	404,094
*Rhipidura cyaniceps*					
NLuzon	Estimated	4.37891	1,572,884	582,549	393,221
	Lower bound	2.84	1,020,114	377,820	255,028
	Upper bound	5.727	2,057,112	761,893	514,278
	Median	4.339	1,558,548	577,240	389,637
Bicol Peninsula	Estimated	0.55273	198,538	73,532	49,639
	Lower bound	0	0	0	0
	Upper bound	1.523	547,054	202,612	136,763
	Median	0.581	208,692	77,293	52,173

Mitochondrial haplotype networks (Figs 3, 4, and 5) for the three species reflected the same population structure revealed by the phylogenetic analyses, in which high (in *R*. *cyaniceps* and *C*. *luzoniensis*, 40 and 41 mutational steps respectively) to low (3 in *P*. *cebuensis*) numbers of mutational steps separated Greater Negros-Panay populations from Luzon populations. Luzon birds were grouped in two different haplotype clusters in *R*. *cyaniceps* and *C*. *luzoniensis*, in which 19 and 7 mutational steps, respectively, separated the Bicol Peninsula clades from Northern Luzon clades. Haplotype networks in the three species showed a star-like pattern, suggesting historical demographic expansion after isolation [3]. Apparent signs of secondary contact for *C*. *luzoniensis* were observed in the Mingan Mountains in eastern Luzon, where analyses showed representatives of north and south haplotype groups. One of the haplotypes nested with the Bicol peninsula samples, the other nested with Northern Luzon samples (Fig 4).

## Discussion

The observation that endemic species’ distributions in the Philippines corresponded to biogeographic subprovinces produced a paradigm that dominated biogeographic and taxonomic research for over 25 years, in which repeated cycles of connection and isolation within different PAICs occurred in concert with climatic shifts and sea level change in the Pleistocene, promoting biotic evolution in the archipelago (reviewed in [33]). Although the PAIC paradigm provides a functional explanation for biotic evolution in the Philippine archipelago, molecular analyses have suggested that the this paradigm only explains a portion of diversity patterns in the archipelago, highlighting mixed models as potentially better explanations [18, 33, 39, 54].

Genetic differentiation in the three lowland passerine species in this study also corresponds to a mixed model. Consistent with the PAIC paradigm (H_0_), phylogenetic trees of the three species showed monophyletic sister groups in Greater Luzon and Greater Negros-Panay PAIC. The pattern departs from strict PAIC expectation within Luzon, with substantial structure discovered in this clade in all three species (H_2_). Populations in each of the species showed levels of genetic divergence higher than those proposed for the recognition of full species [11, 63, 101, 102], except for *P*. *cebuensis*. Genetic differentiation within Luzon is particularly evident in *R*. *cyaniceps* and *C*. *luzoniensis*, where high *Fst* values are coupled with a low estimated number of migrants, suggesting the existence of a past barrier to gene flow within the island. Additionally, the association of samples from the Zambales region either with northern Luzon (in *R*. *cyaniceps* and *P*. *cebuensis*), or the Bicol Peninsula, as in *C*. *luzoniensis* suggests that this region in western Luzon has had a dynamic biotic history, in which lowlands may have connected at different times with Northern Luzon or the Bicol Peninsula lowland rainforests.

Following the premises of the PAIC paradigm, most attention on bird differentiation in the Philippines has been devoted to patterns among PAICs [29]. In contrast, within-island differentiation has received relatively little attention [59, 103, 104, 105]. Phylogeographic results in this study showed that genetic differentiation also has occurred within the limits of Greater Luzon PAIC, as in the Zambales region, where phylogenetic patterns suggest a dynamic history, with populations of different species having close relationships to populations on opposite ends of the island.

All of the estimated dates and confidence intervals for population expansion suggest that these events occurred throughout the Pleistocene, spanning several proposed glacial cycles [106], corresponding with models of rainforests expansion and contraction during glacial periods in southeastern Asia [52]. Evidence for demographic expansion in populations of the three species support a scenario in which isolation occurred in different refugia located in Northern Luzon for *R*. *cyaniceps* and *P*. *cebuensis* and in the Bicol Peninsula for *C*. *luzoniensis* and *R*. *cyaniceps*. In the case of *C*. *luzoniensis*, population expansion is further supported by secondary contact between the two distinct phylogroups in the Mingan Mountains, in eastern Luzon.

Considered together, intra-PAIC differentiation patterns and evidence of demographic expansion suggest that populations of some species were fragmented during at least some Pleistocene climatic cycles [49, 59]. A recent study, however, suggested that lowland rainforest connectivity in the Philippines increased during the Pleistocene Last Glacial (LGM, [53]). Although forest may have been widespread in the LGM, differentiation and even population expansion in the focal species likely predated the onset of that period in the three, thus suggesting the main effect of lowland rainforest connectivity was probably biotic redistribution. Although forest connectivity may have increased during glacial periods in some regions [53], the structure and communities of these lowland rainforests may have been different from present-day communities [107, 108], rendering the habitats unsuitable, which may support that populations of the three focal species in Luzon have maintained their differentiation due to ecological vicariance [43, 55]. Environmental niche modelling research has shown that response to environmental change is species-specific [109, 110] and that modifications in some of the abiotic variables may influence distributional patterns, which in turn may have promoted genetic divergence [54]. Also, despite forest connectivity, genetic distances and Fst values for the focal species closely resemble the patterns detected in montane forest taxa [59, 103, 105], for which geographic isolation has played a key role (reviewed in [111]).

Taken together, phylogenetic and phylogeographic patterns in these lowland rainforest endemic birds point to a more dynamic evolution of the Philippine archipelago biota than predicted by the PAIC paradigm. For the three species in this study, a model incorporating the effects of sea level change and climatic changes seems adequate to explain historical demographic patterns. Although sea level changes may have functioned as barriers promoting divergence between PAIC populations, habitat isolation due to climatic changes may have restricted (and perhaps reinforced in different glacial cycles) gene flow in populations within the same PAIC [42, 54]. Results in this study and other studies have revealed that diversity in the Philippines is not only structured between PAICs, but also within PAICs. This result is consistent with recent studies in mammals and reptiles (e.g. [39, 42, 46]), and birds [54, 54, 59, 101, 103, 105, 112].

Our study adds to the growing body of evidence showing that speciation and genetic differentiation may occur within a single island, probably as a consequence of the Pleistocene climatic changes. Recent studies for birds (reviewed in [21]) and mammals in Borneo (e.g. [113]) shown that speciation and differentiation may be more common than expected, either in land-bridge islands in the Sunda Shelf, or in oceanic islands, as in the Philippine archipelago [36, 37, 38, 39, 40, 42, 43, 46, 104, 105, this study], where lowland differentiation in birds is widespread within the two largest Philippine islands of Luzon [54, 104] and Mindanao [54], yet it remains undocumented in smaller islands.

### Taxonomy

Results in this and other studies have repeatedly suggested that the current taxonomy for the Philippine biota does not accurately reflect diversity in the archipelago, which means that current species diversity estimates are better interpreted as conservative [114, 115]. Birds have been long considered as a group with a relatively good taxonomic knowledge; however, the application of the biological species concept in allopatric contexts, such as the Philippine archipelago, has been controversial because of difficult inferences about reproductive isolation between geographically isolated populations [116].

Phylogenetic patterns showed two main clades: one grouped all of the Greater Luzon samples; the other grouped all of the Greater Negros-Panay samples. High genetic distances and Fst values indicate substantial differentiation between these populations in all focal species, with the exception of *P*. *cebuensis* (1.3% average). Populations of the other two taxa in Greater Negros-Panay both show deep genetic differentiation from Luzon populations and diagnostic characters that allow them to be recognized as full species [20, 55].

Additional genetic variation was also discovered within the bounds of a single PAIC. In Greater Luzon PAIC, phylogenetic analyses showed two well-supported monophyletic sister groups. This arrangement apparently agrees with current taxonomy, as there are two recognized subspecies for each taxon [29, 57]; however, geographic ranges between recognized subspecies and phylogeographic groups do not match. In the case of *R*. *cyaniceps*, the two recognized subspecies *cyaniceps* and *pinicola* were included in the same clade, suggesting that morphological variation may be clinal [117] and even ecological, as *pinicola* is mainly restricted to pine forests [29, 117]. The second clade includes all of our samples from the Bicol Peninsula. An average genetic differentiation of 3% (range 2.9–3.1%), and high Fst values (average 0.9, range 0.85–0.95) between the two groups suggests a long period of genetic isolation. Disagreement between current taxonomy and phylogeographic groups was also found in *C*. *luzoniensis*. Samples from Luzon Island were included in two sister clades, with populations from northern Luzon sister to those from western, central, and southern Luzon (Bicol Peninsula), and Catanduanes Island. Deep genetic divergence (average 2.9%, range 2.4–3.3%) and high Fst values (average 0.72, range 0.71–0.73) also suggest long isolation and consequent differentiation. Finally, the only case where taxonomy apparently agrees with our work is in *P*. *cebuensis*. The two recognized subspecies *luzoniensis* and *sorsogonensis* seem to match the monophyletic groups obtained; this subespecific arrangement is supported by a low genetic differentiation (average 0.3%, range 0.3–0.4%) but high Fst values (average 0.86, range 0.7–1).

Throughout the northern Philippines, molecular studies in birds have found values of genetic divergence ranging from 2.7% to 13.8% between populations of the same taxon, suggesting that species boundaries in a number of avian species should be revised [11, 19, 55, 105, 118, 119]. These values bracket the genetic divergence found in the two clades of *R*. *cyaniceps* and *C*. *luzoniensis* found in Luzon Island. It has been suggested that evidence from a single dataset may not be enough, underscoring the need for additional evidence such as morphological and song characters [120, 121]. In the focal species, morphological differentiation is evident when comparing Greater Luzon and Greater Panay Negros lineages, as each has diagnostic characters. However, this task is complicated when comparing lineages within Luzon, as they look almost identical at first sight. However, a cursory inspection in *C*. *luzoniensis* revealed that birds from Bicol and the Polillo islands differ from the northern Luzon birds in the size of the white spots on the undertail (a trait that allowed the recognition of the Polillo taxon *parvimaculatus*), suggesting that diagnostic characters are present. In *R*. *cyaniceps*, birds from the Bicol Peninsula apparently have darker plumage [116]. This situation is reversed in *P*. *cebuensis*, which has the lowest genetic divergence but clear diagnosable characters; subspecies in Luzon differ in the amount and brightness of yellow in the throat and undertail coverts, being brighter in the Bicol Peninsula *sorsogonensis* [122].

Species delimitation may be controversial due to different philosophies and species concept applicability [123]. This may be even more complicated when genetic divergence is not accompanied by clear morphological difference and when this divergence has occurred within the same island e. g. [124]. However, genetic differentiation may occur without corresponding morphological differentiation [125], and recent work in the Philippine archipelago has demonstrated that speciation has occurred within a single island [36, 37, 38, 39, 40, 42, 43, 46, 104, 105]. Whatever philosophy is applied, genetic differentiation and speciation has occurred within a single PAIC, thus deviating from classic PAIC differentiation expectations.

## Supporting Information

S1 Table Specimens, localities and Genbank reference numbers used in this study. (DOCX)

S2 Table Sequence characteristics for each gene partition and the selected models of evolution. (DOCX)

## References

[1] Bermingham E, Moritz C (1998) Comparative phylogeography: concepts and applications. Mol Ecol 7: 367–369.

[2] Templeton AR (1998) Nested clade analyses of phylogeographic data: testing hypotheses about gene flow and population history. Mol Ecol 7: 381–397. 9627999 10.1046/j.1365-294x.1998.00308.x

[3] Avise JC (2000) Phylogeography: the history and formation of species. Harvard University Press.

[4] Arbogast BS, Kenagy GJ (2008) Comparative phylogeography as an integrative approach to historical biogeography. J Biogeogr 28: 819–825.

[5] Mayr E, O’Hara RJ (1986) The biogeographic evidence supporting the Pleistocene forest refugia hypothesis. Evolution 40: 55–67. 28564118 10.1111/j.1558-5646.1986.tb05717.x

[6] Roy MS, Sponer R, Fjeldså J (2001) Molecular systematic and evolutionary history of akalats (Genus Sheppardia): a pre-Pleistocene radiation in a group of African forest birds. Mol Phylogenet Evol 18: 74–83. 11161744 10.1006/mpev.2000.0862

[7] Fjeldså J., Johansson US, Lokugalappatti LGS, Bowie RCK (2007) Diversification of African greenbuls in space and time: linking ecological and historical processes. J Ornithol 148: 359–367.

[8] Miura GJ, Edwards SV (2001) Cryptic differentiation and geographic variation in genetic diversity of Hall’s babbler *Pomatostomus halli* . J Avian Biol 32: 102–110.

[9] Marks BD, Hackett SJ, Capparella AP (2002) Historical relationships among Neotropical lowland forest areas of endemism as determined by mitochondrial DNA sequence variation within the Wedge-billed Woodcreeper (Aves: Dendrocolaptidae: *Glyphorhynchus spirurus*). Mol Phylogenet Evol 24: 153–167. 12128035 10.1016/s1055-7903(02)00233-6

[10] Cheviron ZA, Hackett SJ, Capparella AP (2005) Complex evolutionary history of a Neotropical lowland forest bird (*Lepidothrix coronata*) and its implications for historical hypotheses of the origin of Neotropical avian diversity. Mol Phylogenet Evol 36: 338–357. 15955514 10.1016/j.ympev.2005.01.015

[11] Zou FH, Lim HC, Marks BD, Moyle RG, Sheldon FH (2007) Molecular phylogenetic analysis of the Grey-checke Fulvetta (*Alcipe morrisonia*) of China and Indochina: a case of remarkable genetic divergence in a “species”. Mol Phylogenet Evol 44: 165–174. 17300964 10.1016/j.ympev.2006.12.004

[12] Schmidt BK, Foster JT, Angher GR, Durrant KL, Fleischer RC (2008) A new species of African forest robin from Gabon (Passeriformes: Muscicapidae: *Stiphrornis*). Zootaxa 1850: 27–42.

[13] Nguembock B, Cibois A, Bowie RCK, Cruaud C, Pasquet E (2009) Phylogeny and biogeography of the genus *Illadopsis* (Passeriformes: Timaliidae) reveal the complexity of diversification in some African taxa. J Avian Biol 40: 113–125.

[14] Marks BD (2010) Are lowland rainforests really evolutionary museums? Phylogeography of the green hylia (*Hylia prasina*) in the Afrotropics. Mol Phylogenet Evol 55: 178–184. 10.1016/j.ympev.2009.10.027 19903532

[15] Toon A, Hughes JM, Joseph L (2010) Multilocus analysis of honeyeaters (Aves: Meliphagidae) highlights spatio-temporal heterogeinity in the influence of biogeographic barriers in the Australian monsoonal zone. Mol Ecol 19: 2980–2994. 10.1111/j.1365-294X.2010.04730.x 20609078

[16] Heaney LR, Walsh JS Jr, Peterson AT (2005) The roles of geological history and colonization abilities in genetic differentiation between mammalian populations in the Philippine archipelago. J Biogeogr 32: 229–247.

[17] Roberts TE (2006) History, ocean channels, and distance determine phylogeographic patterns in three widespread Philippine fruit bats. Mol Ecol 15: 2183–2199. 16780434 10.1111/j.1365-294X.2006.02928.x

[18] Esselstyn JA, Brown RM (2009) The role of repeated sea-level fluctuations in the generation of shrew (Soricidae: *Crocidura*) diversity in the Philippine Archipelago. Mol Phylogenet Evol 53: 171–181. 10.1016/j.ympev.2009.05.034 19501180

[19] Sheldon FH, Lohman DJ, Lim HC, Zou F, Goodman SM, Prawiradilaga DM, Winker K, Braile TM, Moyle RG (2009) Phylogeography of the magpie‐robin species complex (Aves: Turdidae: *Copsychus*) reveals a Philippine species, an interesting isolating barrier and unusual dispersal patterns in the Indian Ocean and Southeast Asia. J Biogeogr 36: 1070–1083.

[20] Lim HC, Zou F, Taylor FS, Marks BD, Moyle RG, Voelker G, Shledon FH (2010) Phylogeny of magpie-robins and shamas (Aves: Turdidae: *Copsychus* and *Trichixos*): implications for island biogeography in Southeast Asia. J Biogeogr 37: 1894–1906.

[21] Sheldon FH, Lim HC, Moyle RG (2015) Return to the Malay Archipelago: the biogeography of Sundaic rainforest birds. J Ornithol: 1–23.

[22] Brown RM, Siler CD, Oliveros CH, Esselstyn JA, Diesmos AC, Hosner PA, Linkem CW, Barley AJ, Oaks JR, Sanguila MB, Welton LJ, Blackburn DC, Moyle RG, Peterson AT, Alcala AC (2013) Evolutionary processes of diversification in a model island archipelago. Annu Rev Ecol Evol Syst 44: 411–435.

[23] Ricklefs RE, Bermingham E (2007) The causes of evolutionary radiations in archipelagoes: Passerine birds in the Lesser Antilles. Am Nat 169: 285–297. 10.1086/510730 17230401

[24] Losos JB, Ricklefs RE (2009) Adaptation and diversification on islands. Nature 457: 830–836. 10.1038/nature07893 19212401

[25] Fleischer RC, McIntosh CE, Tarr CL (1998) Evolution on a volcanic conveyor belt: using phylogeographic reconstructions and K-Ar-based ages of the Hawaiian Islands to estimate molecular evolutionary rates. Mol Ecol 7: 533–545. 9628004 10.1046/j.1365-294x.1998.00364.x

[26] Grant PR, Grant BR (2002) Adaptive radiation of Darwin's finches: Recent data help explain how this famous group of Galapagos birds evolved, although gaps in our understanding remain. Am Sci 90: 130–140.

[27] Gillespie R (2004). Community assembly through adaptive radiation in Hawaiian spiders. Science 303: 356–359. 14726588 10.1126/science.1091875

[28] Rabosky DL, Glor RE (2010) Equilibrium speciation dynamics in a model adaptive radiation of island lizards. Proc Natl Acad Sci USA 107: 22178–22183. 10.1073/pnas.1007606107 21135239 PMC3009809

[29] Setiadi MI, McGuire JA, Brown RM, Zubairi M, Iskandar DT, Andayani N, Evans BJ (2011) Adaptive radiation and ecological opportunity in Sulawesi and Philippine fanged frog (*Limnonectes*) communities. Am Nat 178: 221–240. 10.1086/660830 21750386

[30] Dickinson EC, Kennedy RC, Parkes KC (1991) The birds of the Philippines Checklist No. 12. Tring: British Ornithologists’ Union.

[31] Coates BJ, Bishop KD (1997) A guide to the birds of Wallacea. Alderley: Dove Publications.

[32] Kennedy RS, Gonzales PC, Dickinson EC, Miranda HC Jr, Fisher TH (2000) A guide to the birds of the Philippines. Oxford: Oxford University Press.

[33] Diamond JM (1977) Continental and insular speciation in Pacific land birds. Syst Zool 26: 263–268.

[34] Coyne JA, Price TD (2000) Little evidence for sympatric speciation in birds. Evolution 54: 2166–2171. 11209793 10.1111/j.0014-3820.2000.tb01260.x

[35] Brown RM, Guttman SI (2002) Phylogenetic systematic of the *Rana signata* complex of Philippine and Bornean stream frogs: reconsideration of Huxley’s modifications of Wallace’s Line at the Oriental-Australian faunal zone interface. Biol J Linn Soc Lond 76, 393–461.

[36] Sanguila MB, Siler CD, Diesmos AC, Nuñeza O, Brown RM (2011) Phylogeography, geographic structure, genetic variation, and potential species boundaries in Philippine slender toads. Mol Phylogenet Evol 61: 333–350. 10.1016/j.ympev.2011.06.019 21757017

[37] Linkem CW, Hesed KM, Diesmos AC, Brown RM (2010) Species boundaries and cryptic lineage diversity in a Philippine forest skink complex (Reptilia; Squamata; Scincidae: Lygosominae). Mol Phylogenet Evol 56: 572–585. 10.1016/j.ympev.2010.03.043 20403445

[38] Siler CD, Oaks JR, Esselstyn JA, Diesmos AC, Brown RM (2010) Phylogeny and biogeography of Philippine bent-toed geckos (Gekkonidae: *Cyrtodactylus*) contradict a prevailing model of Pleistocene diversification. Mol Phylogenet Evol 55, 699–710. 10.1016/j.ympev.2010.01.027 20132898

[39] Welton LJ, Siler CD, Linkem CW, Diesmos AC, Brown RM (2010) Philippine bent-toed geckos of the *Cyrtodactylus agusanensis* complex: multilocus phylogeny, morphological diversity, and descriptions of three new species. Herpetological Monograph 24: 55–85.

[40] Welton LJ, Travers SL, Siler CD, Brown RM (2014) Integrative taxonomy and phylogeny-based species delimitation of Philippine water monitor lizards (*Varanus salvator* Complex) with descriptions of two new cryptic species. Zootaxa 3881: 201–227. 25543631 10.11646/zootaxa.3881.3.1

[41] Steppan SJ, Zawadzki C, Heaney LR (2003) Molecular phylogeny of the endemic Philippine rodent *Apomys* (Muridae) and the dynamics of diversification in an oceanic archipelago. Biol J Linn Soc Lond 80: 699–715.

[42] Heaney LR, Balete DS, Rickart EA, Alviola PA, Duya MRM, Duya MV, Veluz MJ, Vandevrede L, Steppan SJ (2011) Chapter 1: Seven New Species and a New Subgenus of Forest Mice (Rodentia: Muridae: *Apomys*) from Luzon Island. Fieldiana Life Earth Sci: 1–60.

[43] Justiniano R, Schenk JJ, Balete DS, Rickart EA, Esselstyn JA, Heaney LR, Steppan SJ (2015) Testing diversification models of endemic Philippine forest mice (*Apomys*) with nuclear phylogenies across elevational gradients reveals repeated colonization of isolated mountain ranges. J Biogeogr 42: 51–64.

[44] Mayr E, Diamond JE (2001) The birds of northern Melanesia: Speciation, ecology, and biogeography. New York: Oxford University Press.

[45] Milá B, Warren BH, Heeb P, Thébaud C (2010) The geographic scale of diversification on islands: genetic and morphological divergence at a very small spatial scale in the Mascarene grey white-eye (Aves: *Zosterops borbonicus*). BMC Evol Biol 10: 158 10.1186/1471-2148-10-158 20504327 PMC2894829

[46] Esselstyn JA, Oliveros CH, Moyle RG, Peterson AT, McGuire JA, Brown RM (2010) Integrating phylogenetic and taxonomic evidence illuminates complex biogeographic patterns along Huxley’s modification of Wallace’s Line. J Biogeogr 37: 2054–2066.

[47] Hall R (2002) Cenozoic geological and plate tectonic evolution of SE Asia and the SW Pacific: computer-based reconstructions and animations. J Asian Earth Sci. 20: 353–434.

[48] Heaney LR (1985) Zoogeographic evidence for middle and late Pleistocene land bridges to the Philippine islands. Modern Quaternary Research in Southeast Asia 9: 127–144.

[49] Brown RM, Diesmos AC (2002) Application of lineage-based species concepts to oceanic islands frog populations: the effects of differing philosophies on the estimation of Philippine biodiversity. Silliman Journal 42, 133–162.

[50] Voris HK (2000) Maps of the Pleistocene sea levels in South East Asia: shorelines, river systems, time durations. J Biogeogr 27: 1153–1167.

[51] Bird MI, Taylor D, Hunt C (2005) Palaeoenvironments of insular Southeast Asia during the Last Glacial Period: a savanna corridor in Sundaland? Quat Sci Rev 24: 2228–2242.

[52] Cannon CH, Morley RJ, Bush ABG (2009) The current refugial rainforests of Sundaland are unrepresentative of their biogeographic past and highly vulnerable to disturbance. Proc Natl Acad Sci USA 106: 11188–11193. 10.1073/pnas.0809865106 19549829 PMC2708749

[53] Peterson AT, Ammann CM (2013) Global patterns of connectivity and isolation of populations of forest bird species in the late Pleistocene. Glob Ecol Biogeogr 22: 596–606.

[54] Hosner PA, Sánchez-González LA, Peterson AT, Moyle RG (2014) Climate-driven diversification and Pleistocene refugia in Philippine birds: evidence from phylogeographic structure and paleo-environmental niche modeling. Evolution 68: 2658–2674. 10.1111/evo.12459 24890129

[55] Sánchez-González LA, Moyle RG (2011) Molecular systematics and species limits in the Philippine fantails (Aves: *Rhipidura*). Mol Phylogenet Evol 61: 290–299. 10.1016/j.ympev.2011.06.013 21722744

[56] Nyári A, Benz BW, Jønsson KA, Fjeldså J, Moyle RG (2009) Phylogenetic relationships of fantails (Aves: Rhipiduridae). Zool Scr 2009: 1–9.

[57] Gilliard ET (1950) Notes on a collection of birds from Bataan, Luzon, Philippine Islands. Bull Amer Mus Nat Hist 94: 459–504.

[58] Delacour J, Mayr E (1946) Birds of the Philippines. New York: The MacMillan Company.

[59] Jones AW, Kennedy RS (2008) Evolution in a tropical archipelago; comparative phylogeography of Philippine fauna and flora reveals complex patterns of colonization and diversification. Biol J Linn Soc Lond 95: 620–639.

[60] Olsson U, Alstrom P, Ericson PGP, Sundberg P (2005) Non-monophyletic taxa and cryptic species: Evidence from a molecular phylogeny of leaf-warblers (*Phylloscopus*, Aves). Mol Phylogenet Evol 36: 261–276. 15955509 10.1016/j.ympev.2005.01.012

[61] Sorenson MD, Ast JC, Dimcheff DE, Yuri T, Mindell DP (1999) Primers for a PCR-based approach to mitochondrial genome sequencing in birds and other vertebrates. Mol Phylogenet Evol 12: 105–114. 10381314 10.1006/mpev.1998.0602

[62] Chesser RT (1999) Molecular systematics of the rhinocryptid genus *Pteroptochos* . Condor 101, 439–446.

[63] Sheldon FH, Whittingham LA, Moyle RG, Slikas B, Winkler DW (2005) Phylogeny of swallows (Aves: Hirundinidae) estimated from nuclear and mitochondrial DNA sequences. Mol Phylogenet Evol 35: 254–270. 15737595 10.1016/j.ympev.2004.11.008

[64] Marini M, Hackett SJ (2002) A multifaceted approach to the characterization of an intergeneric hybrid manakin (Pipridae) from Brazil. Auk 119: 1114–1120.

[65] Kimball RT, Braun EL, Barker FK, Bowie RCK, Braun MJ, Chojnowski JL, Hackett SJ, Han K-L, Harshman J, Heimer-Torres V, Holznagel W, Huddleston CJ, Marks BD, Miglia KJ, Moore WS, Reddy S, Sheldon FH, Smith JV, Witt CC, Yuri T (2009) A well-tested set of primers to amplify regions spread across the avian genome. Mol Phylogenet Evol 50: 654–660. 10.1016/j.ympev.2008.11.018 19084073

[66] Van Tuinen M, Butvill DV, Kirsch JAW, Hedges SB (2001) Convergence and divergence in the evolution of aquatic birds. Proc R Soc Lond B 268: 1345–1350. 10.1098/rspb.2001.1679 PMC1088747 11429133

[67] Slade RW, Moritz C, Heideman A, Hale PT (1993) Rapid assessment of single-copy nuclear DNA variation in diverse species. Mol Ecol 2: 359–373. 7909260 10.1111/j.1365-294x.1993.tb00029.x

[68] Heslewood MM, Elphinstne MS, Tidemann SC, Baverstock PR (1998) Myoglobin intron variation in the Gouldian Finch *Erytrhura gouldiae* assessed by temperature gradient gel electrophoresis. Electrophoresis 19: 142–151. 9548272 10.1002/elps.1150190203

[69] Katoh K, Kuma KI, Toh H, Miyata T (2005) MAFFT version 5: improvement in accuracy of multiple sequence alignment. Nucleic Acids Res 33: 511–518. 15661851 10.1093/nar/gki198 PMC548345

[70] Geneious version 7.0.2. created by Biomatters. Available from http://www.geneious.com/

[71] Stamatakis A (2006) RAxML-VI—HPC: Maximum Likelihood-based phylogenetic analyses with thousands of taxa and mixed models. Bioinformatics 22: 2688–2690. 16928733 10.1093/bioinformatics/btl446

[72] Felsenstein J (1985) Confidence limits on phylogenies: An approach using the bootstrap. Evolution 39: 783–791. 28561359 10.1111/j.1558-5646.1985.tb00420.x

[73] Ronquist F, Teslenko M, van der Mark P, Ayres DL, Darling A, Hohna S, Larget B, Liu L, Suchard MA, Huelsenbeck JP (2012) MrBayes 3.2: efficient Bayesian phylogenetic inference and model choice across a large model space. Syst Biol 61: 539–542. 10.1093/sysbio/sys029 22357727 PMC3329765

[74] Bull JJ, Huelsenbeck JP, Cunnigham CW, Swofford DL, Waddell PJ (1993) Partitioning and combining data in phylogenetic analysis. Syst Biol 42: 384–397.

[75] Brandley MC, Schmitz A, Reeder TW (2005) Partitioned Bayesian analysis, partition choice, and the phylogenetic relationships of lizards. Syst Biol 54, 373–390. 16012105 10.1080/10635150590946808

[76] Nylander JAA (2004). MrModeltest v2. Program distributed by the author. Evolutionary Biology Centre, Uppsala University.

[77] Rambaut A, Drummond AJ (2007) Tracer 1.5.0. Available from http://beast.bio.ed.ac.uk/Tracer

[78] Wilgenbush JC, Warren DL, Swofford DL (2004) AWTY: A system for graphical exploration of MCMC convergence in Bayesian phylogenetic inference. http://ceb.csit.fsu.edu/awty. 10.1093/bioinformatics/btm388 17766271

[79] Librado P, Rozas J (2009) DnaSP v5: A software for comprehensive analysis of DNA polymorphism data. Bioinformatics 25: 1451–1452. 10.1093/bioinformatics/btp187 19346325

[80] Nei M (1987) Molecular evolutionary genetics. New York: Columbia Univ. Press.

[81] Collar NJ (2011) Species limits in some Philippine birds including the Greater Flameback *Chrysocolaptes lucidus* . Forktail 27: 29–38.

[82] Excoffier L, Lischer HEL (2010) Arlequin suite ver 3.5: A new series of programs to perform population genetics analyses under Linux and Windows. Mol Ecol Res 10: 564–567. 10.1111/j.1755-0998.2010.02847.x 21565059

[83] Hartl DL, Clark AG (1997) Principles of population genetics. Sunderland: Sinauer Associated Inc.

[84] Fu XY (1997) Statistical tests of neutrality of mutations against population growth, hitchhiking, and background selection. Genetics 147: 915–925. 9335623 10.1093/genetics/147.2.915 PMC1208208

[85] Tajima F (1989) The effect of change in population size on DNA polymorphism. Genetics 125: 597–601. 10.1093/genetics/123.3.597 PMC1203832 2599369

[86] Slatkin M, Hudson RR (1992) Pairwise comparisons of mitochondrial DNA sequences in stable and exponential growth populations. Genetics 129: 555–562. 10.1093/genetics/129.2.555 PMC1204643 1743491

[87] Rogers AR, Harpending HC (1992) Population growth makes waves in the distribution of pairwise genetic differences. Mol Biol Evol 9: 552–569. 1316531 10.1093/oxfordjournals.molbev.a040727

[88] Ramos-Onsins SE, Rozas J (2002) Statistical properties of newneutrality tests against population growth. Mol Biol Evol 19: 2092–2100. 12446801 10.1093/oxfordjournals.molbev.a004034

[89] Weir JT, Schluter D (2008) Calculating the avian molecular clock. Mol Ecol 17: 2321–2328. 10.1111/j.1365-294X.2008.03742.x 18422932

[90] Arbogast BS, Drovetski SV, Curry RL, Boag PT, Seutin G, Grant PR, Grant R, Anderson DJ (2006) The origin and diversification of Galapagos mockingbirds. Evolution 60: 370–382. 16610327

[91] Ho SYW, Phillips MJ, Cooper A, Drummond AJ (2005) Time dependency of molecular rate estimates and systematic overestimation of recent divergence time. Mol Biol Evol 22: 1561–1568. 15814826 10.1093/molbev/msi145

[92] Lim HC, Rahman MA, Lim SLH, Moyle RG, Sheldon FH (2011) Revisiting Wallace’s haunt: Coalescent simulations and comparative niche modeling reveal historical mechanisms that promoted avian population divergence in the Malay Archipelago. Evolution. 65: 321–334. 10.1111/j.1558-5646.2010.01105.x 20796023

[93] Rogers AR (1995) Genetic evidence for a Pleistocene population explosion. Evolution 49: 608–615. 28565146 10.1111/j.1558-5646.1995.tb02297.x

[94] Schneider S, Excoffier L (1999) Estimation of past demographic parameters from the distribution of pairwise differences when the mutation rates vary among sites: application to human mitochondrial DNA. Genetics, 152, 1079–1089. 10388826 10.1093/genetics/152.3.1079 PMC1460660

[95] Posada D, Crandall KA (2001) Intraspecific gene genealogies: trees grafiting into networks. Trends Ecol Evol 16: 37–45. 11146143 10.1016/s0169-5347(00)02026-7

[96] Bandelt H-J, Forster P, Röhl A (1999) Median-joining networks for inferring intraspecific phylogenies. Mol Biol Evol 16: 37–48 10331250 10.1093/oxfordjournals.molbev.a026036

[97] Zink RM, Barrowclough GF (2008) Mitochondrial DNA under siege in avian phylogeography. Mol Ecol 17: 2107–2121. 10.1111/j.1365-294X.2008.03737.x 18397219

[98] McGregor RC (1909) A manual of Philippine birds. Manila: Bureau of Science.

[99] Grant WS, Bowen BW (1998) Shallow population histories in deep evolutionary lineages of marine fishes: insight from sardines and anchovies and lessons for conservation. J Heredity 89: 415–426.

[100] Hamilton MB (2009) Population genetics. Oxford: Wiley-Blackwell Ltd.

[101] Johns GC, Avise JC (1998) A comparative summary of genetic distances in the vertebrates from the mitochondrial Cytochrome b gene. Mol Biol Evol 15: 1481–1490. 12572611 10.1093/oxfordjournals.molbev.a025875

[102] Whittingham LA, Slikas B, Winkler DW, Sheldon FH (2002) Phylogeny of the tree swallow genus, Tachycineta (Aves: Hirundinidae), by Bayesian analysis of mitochondrial DNA sequences. Mol Phylogenet Evol 22: 430–44. 11884168 10.1006/mpev.2001.1073

[103] Jones AW, Kennedy RS (2008). Plumage convergence and evolutionary history of the island thrush in the Philippines. Condor 110: 35–44.

[104] Hosner PA, Boggess NC, Alviola P, Sánchez-González LA, Oliveros CH, Urriza R, Moyle RG (2013). Phylogeography of the Robsonius Ground-Warblers (Passeriformes: Locustellidae) Reveals an Undescribed Species from Northeastern Luzon, Philippines. Condor 115: 630–639.

[105] Hosner PA, Nyári ÁS, Moyle RG (2013). Water barriers and intra‐island isolation contribute to diversification in the insular *Aethopyga* sunbirds (Aves: Nectariniidae). J Biogeogr, 40: 1094–1106.

[106] Lisiecki LE, Raymo ME (2005) A Pliocene-Pleistocene stack of 57 globally distributed benthic δ^18^O records. Paleoceanography 20: PA1003, 10.1029/2004PA001071

[107] Bush MB, Colinvaux PA (1990) A pollen record of a complete glacial cycle from lowland Panama. J Veg Sci 1:105–118.

[108] Correa-Metrio A, Bush MB, Cabrera KR, Sully S, Brenner M, Hodell DA, Escobar J, Guilderson T (2012) Rapid climate change and no-analog vegetation in lowland Central America during the last 86,000 years. Quat Sci Rev 38: 63–75.

[109] Warren DL, Glor RE, Turelli M (2008) Environmental niche equivalency versus conservatism: quantitative approaches to niche evolution. Evolution 62: 2868–2883. 10.1111/j.1558-5646.2008.00482.x 18752605

[110] Peterson AT, Soberón J, Pearson RG, Anderson RP, Martínez-Meyer E, Nakamura M, Araújo MB (2011) Ecological niches and geographic distributions (MPB-49). Princeton: Princeton University Press.

[111] Fjeldså J, Bowie RCK, Rahbek C (2012) The role of mountain ranges in the diversification of birds. Annu Rev Ecol Evol Syst 43: 249–265.

[112] Sheldon FH, Oliveros CH, Taylor SS, McKay B, Lim HC, Rahman MA, Mays H, Moyle RG (2012) Molecular phylogeny and insular biogeography of the lowland tailorbirds of Southeast Asia (Cisticolidae: *Orthotomus*). Mol Phylogenet Evol 65: 54–63. 10.1016/j.ympev.2012.05.023 22687636

[113] Den Tex RJ, Thorington R, Maldonado JE, Leonard JA (2010) Speciation dynamics in the SE Asian tropics: putting a time perspective on the phylogeny and biogeography of Sundaland tree squirrels, *Sundasciurus* . Mol Phylogenet Evol 55: 711–720. 10.1016/j.ympev.2009.12.023 20040379

[114] Peterson AT (2006) Taxonomy *is* important in conservation: a preliminary reassessment of Philippine species-level bird taxonomy. Bird Conserv Int 16: 155–173.

[115] Lohman DJ, Ingram KK, Prawiradilaga DM, Winker K, Sheldon FH, Moyle RG, Ng PKL, Ong PS, Wang LK, Braile TM, Astuti D, Meier R (2010) Cryptic genetic diversity in “widespread” Southeast Asian bird species suggests that Philippine avian endemism is gravely underestimated. Biol Conserv 143: 1885–1890.

[116] Zink RM, McKitrick MC (1995) The debate over species concepts and its implications for ornithology. Auk 112, 701–719.

[117] Parkes KC (1958) A new race of the blue-headed fantail (*Rhipidura cyaniceps*) from northern Luzon, Philippine Islands. Am Mus Novit: 1891.

[118] Gamauf A, Gjershaug JO, Røv N, Kvaløy K, Haring E (2005) Species or subspecies? The dilemma of taxonomic ranking of some South-East Asian hawk-eagles (genus *Spizaetus*). Bird Conserv Int, 15: 99–117.

[119] Oliveros CH, Moyle RG (2010) Origin and diversification of Philippine bulbuls. Mol Phylogenet Evol 154: 822–832. 10.1016/j.ympev.2009.12.001 19995611

[120] Schlick-Steiner BC, Seifert B, Stauffer C, Christina E, Crozier RH, Steiner FM (2007) Without morphology, cryptic species stay in taxonomic crypsis following discovery. Trends Ecol Evol 22: 391–392. 17573150 10.1016/j.tree.2007.05.004

[121] Winker K (2009) Reuniting phenotype and genotype in biodiversity research. BioScience 59 8: 657–665.

[122] Bairlein F (2006) Family Sylviidae (Old World Warblers) Pp. 492–712. In: Del Hoyo J, Elliot A, Christie DA, editors. Handbook of the birds of the World. Vol. 11. Old World Flycatchers to Old World Warblers. Barcelona: Lynx Edicions.

[123] de Queiroz K (2007) Species concepts and species delimitation. Syst Biol 56: 879–886. 18027281 10.1080/10635150701701083

[124] Lim HC, Chua VL, Benham PM, Oliveros CH, Rahman MA, Moyle RG, Sheldon FH (2014) Divergence history of the Rufous-tailed Tailorbird (*Orthotomus sericeus*) of Sundaland: Implications for the biogeography of Palawan and the taxonomy of island species in general. Auk 131: 629–642.

[125] Barley AJ, White J, Diesmos AC, Brown RM (2013) The challenge of species delimitation at the extremes: diversification without morphological change in Philippine sun skinks. Evolution 67: 3556–3572. 10.1111/evo.12219 24299408

