# Supplementary material for: Genetic Differentiation in Insular Lowland Rainforests: Insights from Historical Demographic Patterns in Philippine Birds
Source: PLoS One. 2015 Aug 27;10(8):e0134284. doi: 10.1371/journal.pone.0134284 (PMC4552387; doi:10.1371/journal.pone.0134284)
Supplement: S1 Table — (DOCX) [file pone.0134284.s001.docx]

| Table S1 File  Taxa included in this study and Genbank Accesion Numbers | | | | | | | |
| --- | --- | --- | --- | --- | --- | --- | --- |
| **Species** | **Tissue/Cat** | **Source^a^** | **Locality (Island)** | **Genbank Accession Number** | | | |
|  |  |  |  | **ND3** | **ND2** | **GAPDH** | **MYO** |
| *Rhipidura cyaniceps* | 17952 | KUNHM | Luzon, Mt Labo | KT162960 | KT371021 | − | − |
| *R. cyaniceps* | 17958 | KUNHM | Luzon, Mt Labo | KT162961 | KT371041 | − | − |
| *R. cyaniceps* | 17959 | KUNHM | Luzon, Mt Labo | KT162962 | KT371022 | − | − |
| *R. cyaniceps* | 17960 | KUNHM | Luzon, Mt Labo | KT162963 | KT371040 | − | − |
| *R. cyaniceps* | 19380 | KUNHM | Luzon, Casiguran | KT162964 | KT371023 | − | − |
| *R. cyaniceps* | 19633 | KUNHM | Luzon, Casiguran | KT162965 | KT371024 | − | − |
| *R. cyaniceps* | 19637 | KUNHM | Luzon, Casiguran | KT162966 | KT371025 | − | − |
| *R. cyaniceps* | 20179 | KUNHM | Luzon, Aurora | KT162967 | KT371026 | − | − |
| *R. cyaniceps* | 20183 | KUNHM | Luzon, Aurora | KT162968 | KT371027 | − | − |
| *R. cyaniceps* | 20184 | KUNHM | Luzon, Aurora | KT162969 | KT371028 | − | − |
| *R. cyaniceps* | 20204 | KUNHM | Luzon, Aurora | KT162970 | KT371029 | − | − |
| *R. cyaniceps* | 20206 | KUNHM | Luzon, Aurora | KT162971 | KT371030 | − | − |
| *R. cyaniceps* | 21050 | KUNHM | Luzon, Mt Palali | KT162973 | KT371031 | − | − |
| *R. cyaniceps* | 21053 | KUNHM | Luzon, Mt Palali | KT162974 | KT371032 | − | − |
| *R. cyaniceps* | 21054 | KUNHM | Luzon, Mt Palali | KT162975 | KT371033 | − | − |
| *R. cyaniceps* | 21065 | KUNHM | Luzon, Mt Palali | KT162976 | KT371034 | − | − |
| *R. cyaniceps* | 21068 | KUNHM | Luzon, Mt Palali | KT162977 | KT371035 | − | − |
| *R. cyaniceps* | 21080 | KUNHM | Luzon, Mt Palali | KT162978 | KT371042 | − | − |
| *R. cyaniceps* | 454944 | FMNH | Luzon, Mt Amnyao | KT162979 | KT371036 | − | − |
| *R. cyaniceps* | B106 | CMC | Luzon, Zambales | KT162980 | KT371037 | − | − |
| *R. cyaniceps* | B35909 | CMC | Luzon, Mt Mayon | KT162982 | KT371038 | − | − |
| *R. cyaniceps* | B36597 | CMC | Luzon, Zambales | KT162983 | KT371039 | − | − |
| *Copsychus luzoniensis* | 15805 | KUNHM | Negros, Mt Talinis | KT370957 | − | KT119796 | KT119812 |
| *C. luzoniensis* | 17967 | KUNHM | Luzon, Mt Labo | KT370920 | KT370959 | KT119797 | KT119813 |
| *C. luzoniensis* | 17981 | KUNHM | Luzon, Mt Labo | KT370921 | KT370960 | KT119798 | KT119814 |
| *C. luzoniensis* | 18094 | KUNHM | Luzon, Mt Labo | KT370922 | KT370961 | − | − |
| *C. luzoniensis* | 18095 | KUNHM | Luzon, Mt Labo | KT370923 | KT370962 | − | − |
| *C. luzoniensis* | 19637 | KUNHM | Luzon, Casiguran | KT370924 | KT370963 | KT119799 | KT119815 |
| *C. luzoniensis* | 19376 | KUNHM | Luzon, Casiguran | KT370925 | KT370964 | − | KT119816 |
| *C. luzoniensis* | 19640 | KUNHM | Luzon, San Luis | KT370926 | KT370965 | − | KT119817 |
| *C. luzoniensis* | 20162 | KUNHM | Luzon, San Luis | KT370927 | KT370966 | KT119800 | KT119818 |
| *C. luzoniensis* | 20212 | KUNHM | Luzon, Aurora | KT370929 | KT370967 | KT119801 | KT119819 |
| *C. luzoniensis* | 20228 | KUNHM | Luzon, Aurora | KT370930 | KT370968 | KT119802 | KT119820 |
| *C. luzoniensis* | 20343 | KUNHM | Luzon, Aurora | KT370931 | KT370969 | − | − |
| *C. luzoniensis* | 20344 | KUNHM | Luzon, Aurora | KT370932 | KT370970 | − | − |
| *C. luzoniensis* | 21058 | KUNHM | Luzon, Mt Palali | KT370933 | KT370971 | − | − |
| *C. luzoniensis* | 21075 | KUNHM | Luzon, Mt Palali | KT370934 | KT370972 | − | − |
| *C. luzoniensis* | 21097 | KUNHM | Luzon, Mt Palali | KT370935 | KT370973 | − | − |
| *C. luzoniensis* | 21101 | KUNHM | Luzon, Mt Palali | KT370936 | KT370974 | − | − |
| *C. luzoniensis* | 21108 | KUNHM | Luzon, Mt Palali | KT370937 | KT370975 | KT119803 | KT119821 |
| *C. luzoniensis* | 21110 | KUNHM | Luzon, Mt Palali | KT370938 | KT370976 | − | − |
| *C. luzoniensis* | 21116 | KUNHM | Luzon, Mt Palali | KT370939 | KT370977 | − | − |
| *C. luzoniensis* | 446730 | FMNH | Luzon, Zambales | KT370940 | KT370978 | KT119804 | KT119822 |
| *C. luzoniensis* | 449769 | FMNH | Luzon, Mingan Mts | KT370941 | KT370979 | KT119805 | KT119823 |
| *C. luzoniensis* | 449770 | FMNH | Luzon, Mingan Mts | KT370942 | KT370980 | KT119806 | KT119824 |
| *C. luzoniensis* | 449977 | FMNH | Luzon, Mt Palali | KT370943 | KT370981 | − | − |
| *C. luzoniensis* | 449978 | FMNH | Luzon, Mt Palali | KT370944 | KT370982 | − | − |
| *C. luzoniensis* | 462062 | FMNH | Luzon, Lagonoy | JN45904* | JN545960* | KT119807 | − |
| *C. luzoniensis* | 462034 | FMNH | Luzon, Lagonoy | KT370945 | KT370983 | − | KT119825 |
| *C. luzoniensis* | 462035 | FMNH | Luzon, Lagonoy | KT370946 | KT370984 | − | KT119826 |
| *C. luzoniensis* | B128 | CMC | Luzon, Zambales | KT370949 | KT370987 | − | − |
| *C. luzoniensis* | B158 | CMC | Luzon, Zambales | KT370950 | KT370988 | KT119808 | KT119827 |
| *C. luzoniensis* | B225 | CMC | Luzon, Zambales | KT370951 | KT370989 | KT119809 | KT119828 |
| *C. luzoniensis* | B36551 | CMC | Panay, Antique | KT370952 | KT370990 | − | − |
| *C. luzoniensis* | B36553 | CMC | Panay, Antique | KT370953 | KT370991 | − | KT119829 |
| *C. luzoniensis* | B36925 | CMC | Panay, Antique | KT370954 | KT370992 | KT119810 | KT119830 |
| *C. luzoniensis* | B875 | CMC | Panay, Antique | KT370955 | − | KT119811 | KT119831 |
| *C. luzoniensis* | B900 | CMC | Panay, Antique | KT370956 | KT370993 | − | KT119832 |
| *Phyllscopus cebuensis* | 19606 | KUNHM | Luzon, Casiguran | KT371051 | KT371002 | − | − |
| *P. cebuensis* | 19638 | KUNHM | Luzon, San Luis | KT371050 | KT371001 | − | − |
| *P. cebuensis* | 20195 | KUNHM | Luzon, Aurora | KT371049 | KT371000 | − | − |
| *P. cebuensis* | 20247 | KUNHM | Luzon, Aurora | KT371047 | KT370998 | − | − |
| *P. cebuensis* | 20262 | KUNHM | Luzon, Aurora | KT371045 | KT370996 | KT162930 | KT162945 |
| *P. cebuensis* | 20261 | KUNHM | Luzon, Aurora | KT371046 | KT370997 | KT162931 | KT162946 |
| *P. cebuensis* | 20231 | KUNHM | Luzon, Aurora | KT371048 | KT370999 | − | KT162947 |
| *P. cebuensis* | 20272 | KUNHM | Luzon, Aurora | KT371044 | KT370995 | − | − |
| *P. cebuensis* | 20283 | KUNHM | Luzon, Aurora | KT371043 | KT370994 | − | − |
| *P. cebuensis* | 18067 | KUNHM | Luzon, Mt Labo | KT371052 | KT371003 | KT162932 | KT162948 |
| *P. cebuensis* | 17999 | KUNHM | Luzon, Mt Labo | KT371053 | KT371004 | KT162933 | KT162949 |
| *P. cebuensis* | 16017 | KUNHM | Luzon, Mt Labo | KT371054 | KT371006 | KT162934 | KT162950 |
| *P. cebuensis* | 462047 | FMNH | Luzon, Caramoan | KT371055 | KT371005 | KT162935 | KT162951 |
| *P. cebuensis* | B36576 | FMNH | Luzon, Zambales | KT371056 | − | − | KT162952 |
| *P. cebuensis* | B36575 | FMNH | Luzon, Zambales | KT371057 | KT371017 | KT162936 | KT162953 |
| *P. cebuensis* | B75 | FMNH | Luzon, Zambales | KT371058 | − | KT162937 | KT162954 |
| *P. cebuensis* | 454995 | FNHM | Luzon, Mt Malinao | KT371060 | KT371006 | − | KT162955 |
| *P. cebuensis* | 454996 | FNHM | Luzon, Mt Palali | KT371059 | KT371018 | − | − |
| *P. cebuensis* | 449801 | FMNH | Luzon, Mingan Mts | KT371061 | KT371007 | − | KT162956 |
| *P. cebuensis* | 449772 | FMNH | Luzon, Mingan Mts | KT371062 | KT371008 | KT162938 | KT162957 |
| *P. cebuensis* | 21006 | KUNHM | Luzon, Mt Palali | KT371068 | KT371012 | − | − |
| *P. cebuensis* | 21007 | KUNHM | Luzon, Mt Palali | KT371067 | KT371011 | − | − |
| *P. cebuensis* | 21059 | KUNHM | Luzon, Mt Palali | KT371066 | KT371019 | − | − |
| *P. cebuensis* | 21082 | KUNHM | Luzon, Mt Palali | − | KT371009 | KT162939 | KT162958 |
| *P. cebuensis* | 21079 | KUNHM | Luzon, Mt Palali | KT371064 | KT371010 | − | KT162959 |
| *P. cebuensis* | 20880 | KUNHM | Negros, Mt Bungol | KT371069 | KT371013 | KT162940 | − |
| *P. cebuensis* | 20881 | KUNHM | Negros, Mt Bungol | KT371070 | KT371014 | KT162941 | − |
| *P. cebuensis* | 20882 | KUNHM | Negros, Mt Bungol | KT371063 | KT371020 | KT162942 | − |
| *P. cebuensis* | 20883 | KUNHM | Negros, Mt Bungol | KT371072 | KT371015 | KT162943 | − |

^a^Institution abbreviations: CMCM, Cincinnati Museum Center; FMNH, Field Museum of Natural History; KUNHM, Natural History Museum, University of Kansas.

*Secuences obtained in Sánchez-González and Moyle (2011)
