# Supplementary material for: Genetic Differentiation in Insular Lowland Rainforests: Insights from Historical Demographic Patterns in Philippine Birds
Source: PLoS One. 2015 Aug 27;10(8):e0134284. doi: 10.1371/journal.pone.0134284 (PMC4552387; doi:10.1371/journal.pone.0134284)
Supplement: S2 Table — (DOCX) [file pone.0134284.s002.docx]

Table S2. Sequence characteristics and models of evolution selected for the different codon positions in the genes analyzed in the three species studied.

| Taxon | **Gene** | **Length (bp)** | **Parsimony informative** | **Parsimony-uniformative** | **Codon Partition** | **Model selected** |
| --- | --- | --- | --- | --- | --- | --- |
| *Copsychus luzoniensis* | ND3 | 351 | 32 | 6 | 1st codon  2nd codon  3rd codon | K80  F81  HKY + I |
|  | ND2 | 1041 | 155 | 49 | 1st codon  2nd codon  3rd codon | HKY + I  HKY + I  GTR + G |
|  | G3PDH | 265 | 2 | 13 |  | F81 |
|  | Myioglobin | 541 | 7 | 18 |  | HKY + I |
| *Phylloscopus cebuensis* | ND3 | 351 | 47 | 37 | 1st codon  2nd codon  3rd codon | SYM  HKY  HKY + I |
|  | ND2 | 1041 | 169 | 44 | 1st codon  2nd codon  3rd codon | HKY + I  F81 + I  HKY |
|  | G3PDH | 334 | 10 | 16 |  | HKY + I |
|  | Myioglobin | 703 | 14 | 20 |  | JC |
| *Rhipidura cyaniceps* | ND3 | 351 | 110 | 54 | 1st codon  2nd codon  3rd codon | K80 + G  HKY + G  HKY + G |
|  | ND2 | 1041 | 353 | 93 | 1st codon  2nd codon  3rd codon | GTR + G  GTR + I  GTR + I + G |
|  | Fib5 | 575 | 60 | 67 |  | GTR + G |
